# Cerebrovascular vulnerability and fibrosis in human brain aneurysms

**DOI:** 10.1038/s41593-026-02326-9

**Published:** 2026-06-10

**Authors:** Jerry C. Wang, Chang N. Kim, Shubhang Bhalla, Lea Scherschinski, Adnan Gopinadhan, Santhosh Arul, Damian Sanchez, Tyler D. Schriber, Amanda C. M. Apolonio, Belda Gülsuyu, Muhammet M. Öztürk, John P. Andrews, Joseph Kim, Behnam Rezai Jahromi, Mika Niemelä, Martin Lehecka, Aunoy Poddar, Thomas Wälchli, Joshua S. Catapano, Rajeev D. Sen, Michael R. Levitt, Daniel L. Cooke, Kazim Narsinh, Ruchira M. Jha, Tomoki Hashimoto, S. Paul Oh, Eric J. Huang, Edward F. Chang, Daniel A. Lim, Adib A. Abla, Andrew C. Yang, Tomasz J. Nowakowski, Michael T. Lawton, Ethan A. Winkler

**Affiliations:** 1 Department of Neurological Surgery, University of California, San Francisco, San Francisco, CA USA; 2 Eli and Edythe Broad Center for Regeneration Medicine and Stem Cell Research, University of California, San Francisco, San Francisco, CA USA; 3 Department of Anatomy, University of California, San Francisco, San Francisco, CA USA; 4 Department of Psychiatry, University of California, San Francisco, San Francisco, CA USA; 5 Department of Neurosurgery, Barrow Neurological Institute & St Joseph’s Hospital and Medical Center, Phoenix, AZ USA; 6 Barrow Aneurysm and AVM Research Center, Department of Translational Neuroscience, Barrow Neurological Institute, St Joseph’s Hospital and Medical Center, Phoenix, AZ USA; 7 Gladstone Institute of Neurological Disease, Gladstone Institutes, San Francisco, CA USA; 8 Department of Neurology, University of California, San Francisco, San Francisco, CA USA; 9 Department of Neurosurgery, Helsinki University Hospital and University of Helsinki, Helsinki, Finland; 10 Brain Vasculature and Perivascular Niche Laboratory, Department of Oncology, University College London Cancer Institute, University College London, London, UK; 11 Victor Horsley Department of Neurosurgery, National Hospital for Neurology and Neurosurgery, University College London Hospitals NHS Trust, Queen Square, London, UK; 12 Departments of Neurological Surgery, Radiology, Neurology, Mechanical Engineering, and Stroke & Applied Neuroscience Center, University of Washington, Seattle, WA USA; 13 Department of Radiology and Biomedical Imaging, University of California, San Francisco, San Francisco, CA USA; 14 Department of Neurology, Barrow Neurological Institute & St Joseph’s Hospital and Medical Center, Phoenix, AZ USA; 15 Department of Pathology, University of California, San Francisco, San Francisco, CA USA

**Keywords:** Blood-brain barrier, Stroke, Cardiovascular biology, Neuroimmunology

## Abstract

Brain aneurysms are a cerebrovascular disease that results in a severe type of stroke. The cell-specific molecular pathology underlying their formation and rupture is unknown. Here we profile 227,663 neurovascular cells, including 52,946 aneurysmal cells, from a total of 14 adult human brain aneurysms and 11 control vessels. Our atlas of human brain aneurysms, as well as cell-resolution spatial transcriptomics, revealed that pathological cerebrovascular remodeling occurs with the loss of structurally supportive smooth muscle cells and the emergence of activated perivascular fibroblasts, which re-populate the vascular wall and express multiple genes linked to aneurysm risk. Fibrotic changes coincide with fibroblast–myeloid cell signaling pathways and an influx of specialized macrophages that are rarely detected in non-aneurysmal cerebrovasculature and that express destabilizing vascular cell programs. Thus, we reveal an unrecognized interplay between cerebrovascular fibrosis and myeloid inflammation during disease progression, substantially advancing our understanding of the cellular drivers and mechanisms underlying this devastating cerebrovascular disease that will inform translational development.

## Abstract (web summary)

A cell and spatial atlas of human brain aneurysms identifies an interaction between scarring fibroblasts and inflammatory macrophages linked to vessel wall remodeling, disease progression and rupture leading to stroke.

## Main

Stroke is the second leading cause of death worldwide^1,2^. Aneurysmal subarachnoid hemorrhage (aSAH) is an especially severe form of stroke, third frequent after ischemic infarction and spontaneous intracerebral hemorrhage. Most often, aSAH results from a saccular aneurysm, a sac-like bulge in the vessel wall, at branch points of cerebral arteries comprising the circle of Willis at the base of the brain^3^. Brain aneurysms occur due to a loss of supportive structures, such as vascular smooth muscle cells or extracellular matrix (ECM) proteins, which can be caused by genetic factors, environmental factors and blood flow patterns^4–6^. Further weakening of the arterial walls can lead to rupture, resulting in bleeding into the brain or the subarachnoid spaces. Molecular profiling of samples from human brain aneurysms has revealed common pathways in extracellular matrix remodeling and inflammation involved in the development of the disease^7,8^; however, the specific molecular changes that occur in different cell types during the progression of human brain aneurysms are not fully understood.

Single-cell and single-nucleus RNA sequencing (scRNA-seq and snRNA-seq, respectively) has recently defined cell type composition in the human cerebrovasculature and defined immune interactions in arteriovenous malformations^9–12^. In this study, we used single-cell and spatial transcriptomics to investigate the changes in cellular composition and transcriptional programs that arise in human brain aneurysms. Our results reveal a previously unrecognized role for activated perivascular fibroblasts and a specialized macrophage population in the progression of aneurysm pathology. This atlas of human brain aneurysms and mechanistic investigations provide important understanding of human brain aneurysms that can inform future translational development.

## Deconstructing human brain aneurysms

**Fig. 1:**
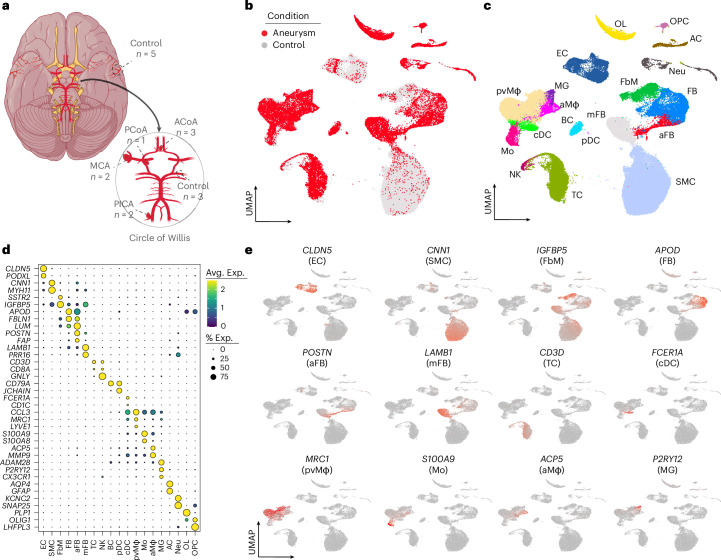
Cell atlas of human brain aneurysms. **a**, Schematic showing location and number of donors for samples profiled with single-cell or single-nucleus sequencing. Inset shows circle of Willis. **b**, Uniform Manifold Approximation and Projection (UMAP) of 100,409 transcriptomes from unruptured brain aneurysms (*n* = 37,560 transcriptomes, 8 donors) and control cerebrovasculature (*n* = 62,849 transcriptomes, 8 donors) colored by condition. **c**, The same as **b** except colored by cell type annotation. **d**, Dot plot showing expression of cell population marker genes. **e**, Expression distribution of cell population marker genes projected on UMAP embeddings from all transcriptomes in **b** and **c**. Gray, low expression; orange–red, high expression; ACoA, anterior communicating artery; PCoA, posterior communicating artery; MCA, middle cerebral artery; PICA, posterior inferior cerebellar artery; EC, endothelial cell; SMC, smooth muscle cell; FbM, fibromyocyte; FB, fibroblast; aFB, activated fibroblast; mFB, myofibroblast; TC, T cell; NK, natural killer cell; BC, B cell; pDC, plasmacytoid dendritic cell; cDC, conventional dendritic cell; pvMϕ, perivascular macrophage; Mo, monocyte; aMϕ, *APC5*^*+*^ macrophage; MG, microglia; AC, astrocyte; Neu, neuron; OL, oligodendrocyte; OPC, oligodendrocyte precursor cell; Avg. Exp., average (mean) expression. Panel **a** created in BioRender; Winkler, E. https://BioRender.com/2atrfp2 (2026).

**Extended Data Fig. 1:**
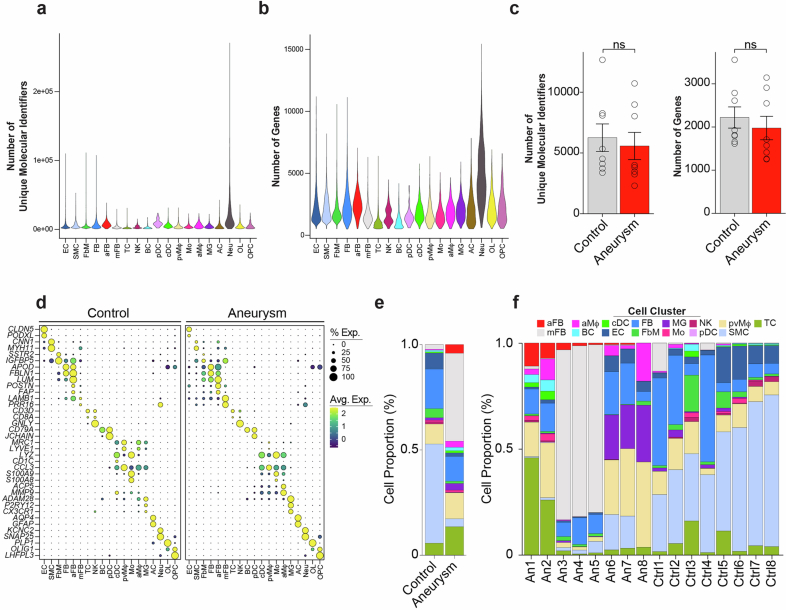
Quality control metrics of cell atlas. **a-b**, Violin plots showing unique molecular identifiers (**a**) and number of genes (**b**) across cell populations after quality filtering. EC, endothelial cell; SMC, smooth muscle cell; FbM, fibromyocyte; FB, fibroblast; aFB, activated fibroblast; mFB, myofibroblast; TC, T-cell; NK, natural killer cell; BC, B-cell; pDC, plasmacytoid dendritic cell; cDC, conventional dendritic cell; pvMϕ, perivascular macrophage; Mo, monocyte; aMϕ, *APC5*^*+*^ macrophage; MG, microglia; AC, astrocyte; Neu, neuron; OL, oligodendrocyte; OPC, oligodendrocyte precursor cell. **c**, Bar graph of number of unique molecular identifiers (left) and number of genes (right) across conditions. Gray, control (*n* = 8 donors); red, aneurysm (n = 8 donors). ns, not significant (two-sided *t*-test; mean ± s.e.m). **d**, Dot plot showing expression of cell population marker genes by condition. Avg., average; Exp., expression; Blue, low expression; yellow, high expression. **e-f**, Bar graph of cell proportion by condition (**e**) and donor (**f**). An., aneurysm; Ctrl., control.

Most brain aneurysms are now treated with endovascular therapy without tissue acquisition^13^, and biopsies from surgical cases are often limited to several milligrams of tissue, thereby challenging applications of single-cell genomics. We leveraged expertise across four high-volume treatment centers and performed scRNA-seq or snRNA-seq on unruptured brain aneurysm tissues isolated from eight patients undergoing neurosurgical treatment (Supplementary Table 1). We used two control tissues based on consensus recommendations^14,15^: surgically acquired pial cortical vasculature (*n* = 5) and postmortem non-aneurysmal circle of Willis arteries (*n* = 3). Fresh tissues were dissociated with established techniques to perform scRNA-seq (*n* = 2 aneurysm and *n* = 5 control donors)^9^. To extend our analyses to a larger cohort of aneurysms, we performed snRNA-seq on fresh frozen aneurysm tissues (*n* = 6 donors) and non-aneurysmal posterior cerebral arteries (*n* = 3 donors) (Fig. 1a). Following quality control (Methods), we recovered 37,560 high-quality aneurysmal transcriptomes (*n* = 21,531 cells; *n* = 16,029 nuclei) from a total of eight aneurysm and eight control donors (Extended Data Fig. 1a–c). Transcriptomes were computationally integrated using Harmony^16^, accounting for differences in sample preparation (Methods), and clustered with control cerebrovascular cells (*n* = 59,520 cells, *n* = 3,329 nuclei) prepared with analogous techniques to guide annotations and facilitate cell-specific comparisons across conditions (*n* = 100,409 transcriptomes) (Fig. 1b)^9^.

**Fig. 2:**
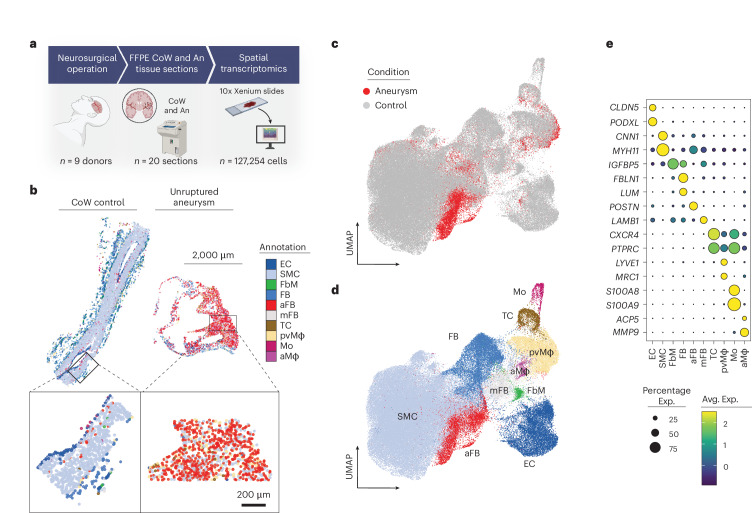
Cell-resolution spatial transcriptomics of human brain aneurysms. **a**, Schematic of the preparation of specimens from the circle of Willis (CoW) controls (*n* = 3 donors) and unruptured brain aneurysms (*n* = 6 donors) for the 10x Genomics Xenium In Situ platform. **b**, Representative spatial plots of segmented cells from control and unruptured aneurysm colored by cell type identity. **c**, UMAP of spatially segmented control and unruptured aneurysm cells. **d**, The same as **c** but color coded by cell type identity. **e**, Dot plot showing expression of cell population marker genes. FFPE, formalin-fixed paraffin-embedded; An, aneurysm. Panel **a** created in BioRender; Winkler, E. https://BioRender.com/wvoa7vj (2026).

**Extended Data Fig. 2:**
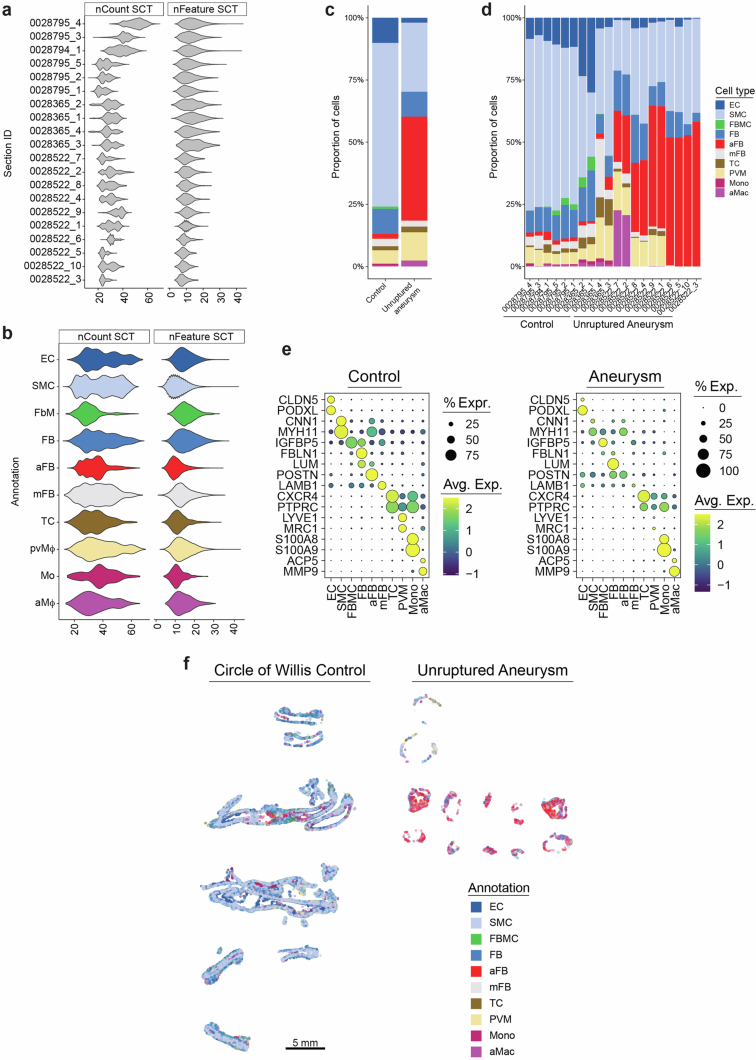
Quality control metrics and spatial cell distributions in unruptured human brain aneurysms using 10x Genomics Xenium *In Situ* Platform. **a**, Violin plots of SCTransformed gene and feature counts across each captured section. **b**, Same as **a** except split across cell type annotation for quality-controlled segmented cells. EC, endothelial cell; SMC, smooth muscle cell; FbM, fibromyocyte; FB, fibroblast; aFB, activated fibroblast; mFB, myofibroblast; TC, T-cell; pvMϕ, perivascular macrophage; Mo, Monocyte; aMϕ, activated perivascular macrophage. **c-d**, Proportion of each cell type annotation by disease status (**c**) and section (**d**). **e**, Dot plot of markers used to annotate each cell type in Circle of Willis control (left) and unruptured aneurysm (right) sections. **f**, Spatial plots of segmented cells from Circle of Willis controls (left) and unruptured aneurysms (right) with each dot colored by cell type identity. All imaged tissue sections shown. Scale bar, 5 mm.

**Extended Data Fig. 3:**
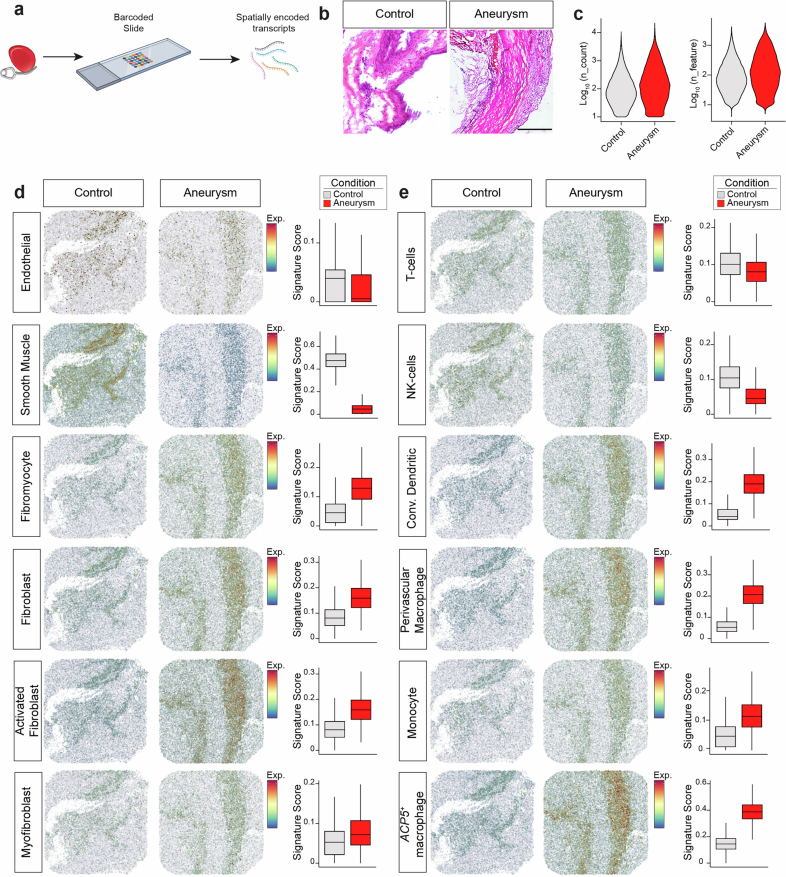
Spatially mapped vascular and immune cell populations in human brain aneurysms using Curio Slide-Seq. **a**, Schematic showing workflow for genome-wide spatial transcriptomic analysis with Slide-seq. **b**, Hematoxylin & eosin stain of control posterior cerebral artery (left) and unruptured middle cerebral artery aneurysm (right) on adjacent sections showing region of analysis. Scale bar, 1 mm. Created in BioRender. Winkler, E. (2026) https://BioRender.com/xro30jr. **c**, Violin plots of unique molecular identifiers (left, n_count) and number of genes (right, n_feature). **d-e**, Spatial feature plot showing expression of cell signature scores in non-aneurysmal posterior cerebral artery (left, n = 33,585 cells from 1 donor) and middle cerebral artery aneurysm (right, n = 32,203 cells from 1 donor). Exp., expression; Red, high expression; Blue, low expression. Adjacent box plots show relative expression by condition. Gray, control; red, aneurysm. Center lines indicate medians; box limits indicate the 25th and 75th percentiles; whiskers extend to the most extreme data points no more than 1.5 × IQR from the box; points beyond the whiskers are shown as outliers.

We identified 19 transcriptionally distinct cell populations, including six cerebrovascular, nine immune and four adjacent glial-neuronal cell populations (Fig. 1c–e, Extended Data Fig. 1d and Supplementary Table 2). Each cell population was represented in multiple donors (Extended Data Fig. 1e, f). Furthermore, cell-resolution spatial transcriptomics using the 10x Genomics Xenium platform, which applies highly multiplexed fluorescent in situ hybridization across hundreds of genes^17^, together with best practices for image-based spatial genomics^18^, was used to orthogonally segment all molecularly defined cerebrovascular cell populations and their perivascular immune niches in independent non-aneurysmal intracranial arteries (*n* = 3 donors, 111,868 segmented cells) and unruptured brain aneurysms (*n* = 6 donors, 15,386 segmented cells) (Fig. 2a–d, Extended Data Fig. 2 and Supplementary Table 1). We spatially localized all six of our previously identified cerebrovascular populations, and four immune cell populations, including both perivascular macrophage populations (Fig. 2e), supporting the ability of this assay to resolve at cell-resolution the identities of vascular and perivascular cell populations found in aneurysms and matching circle of Willis controls. As expected, we did not spatially localize any of the glial-neuronal cell populations nor the remaining five immune cells that are typically found in the brain parenchyma (microglia) or circulation (for example, dendritic cells, B cells, natural killer cells), as the aneurysms and circle of Willis control specimens were largely cleaned of residual parenchyma and blood products before spatial transcriptomic profiling (Methods). Cell segmentation and spatially informed cluster annotations^19^ accurately identified cells whose marker gene expression signatures matched those found in the single-cell analysis (Fig. 2e and Extended Data Fig. 3), confirming that our cellular annotations capture the same transcriptional programs initially defined in the single-cell data. Thus, we provide a cell and spatial atlas of the cell composition of aneurysms in the human brain.

## Vascular cell-type-specific changes in brain aneurysms

**Fig. 3:**
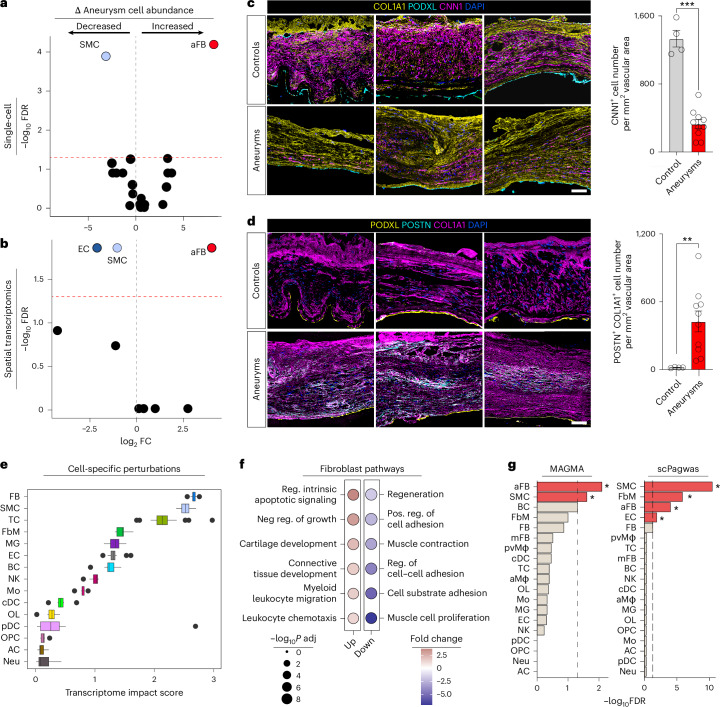
Cerebrovascular cell-specific perturbations in brain aneurysms. **a**, Volcano plot of cell composition changes in brain aneurysms compared with control cerebrovasculature profiled using the cell atlas. Colored cell populations symbolize FDR < 0.05 based on moderated two-sided *t*-test with Benjamini–Hochberg correction using propeller analysis. **b**, The same as **a** except on segmented cells from the cell-resolution Xenium spatial transcriptomics assay. **c**, Left: representative confocal microscopy imaging of PODXL^+^ endothelial cells (cyan), CNN1^+^ smooth muscle cells (magenta), and COL1A1^+^ perivascular fibroblasts (yellow) in control middle cerebral arteries or brain aneurysm. Three representative examples shown. Scale bar, 50 μm. Right: quantification of CNN1^+^ smooth muscle cell number per mm^2^ vascular area. ***P = 0.0003, *n* = 4 control middle cerebral arteries, *n* = 10 aneurysms; two-sided *t-*test; mean ± s.e.m. **d**, Left: representative confocal microscopy imaging of PODXL^+^ endothelial cells (yellow), POSTN^+^ (cyan) and COL1A1^+^ (magenta) perivascular imaging fibroblasts in control middle cerebral arteries or brain aneurysm. Three representative examples shown. Scale bar, 50 μm. Right: quantification of POSTN^+^ and COL1A1^+^ perivascular fibroblasts. **P = 0.0023, *n* = 4 control middle cerebral arteries, *n* = 10 aneurysms; two-sided *t*-test; mean ± s.e.m. **e**, Jack-knifed TRADE (*n* = 15 samples) of conserved cell populations between aneurysms and controls in the cell atlas, with cell types listed in descending median transcriptome impact scores. Center lines indicate medians; box limits indicate the 25th and 75th percentiles; whiskers extend to the most extreme data points no more than 1.5× IQR from the box; points beyond the whiskers are shown as outliers. **f**, Dot plot of enriched biological process in aneurysm perivascular fibroblasts (Bonferroni-corrected *P* value < 0.05, based on single-cell pathway analysis). **g**, Bar graph showing trait-relevant cell populations for MAGMA (left) and scPagwas (right). Red with *Benjamini–Hochberg adjusted *P* value < 0.05 (block bootstrapping deployed for scPagwas); bold, cell types identified in common between MAGMA and scPagwas; dashed line, Benjamini–Hochberg adjusted *P* value cutoff of 0.05. Neg, negative; reg., regulation; adj, adjusted; Pos., positive.

**Extended Data Fig. 4:**
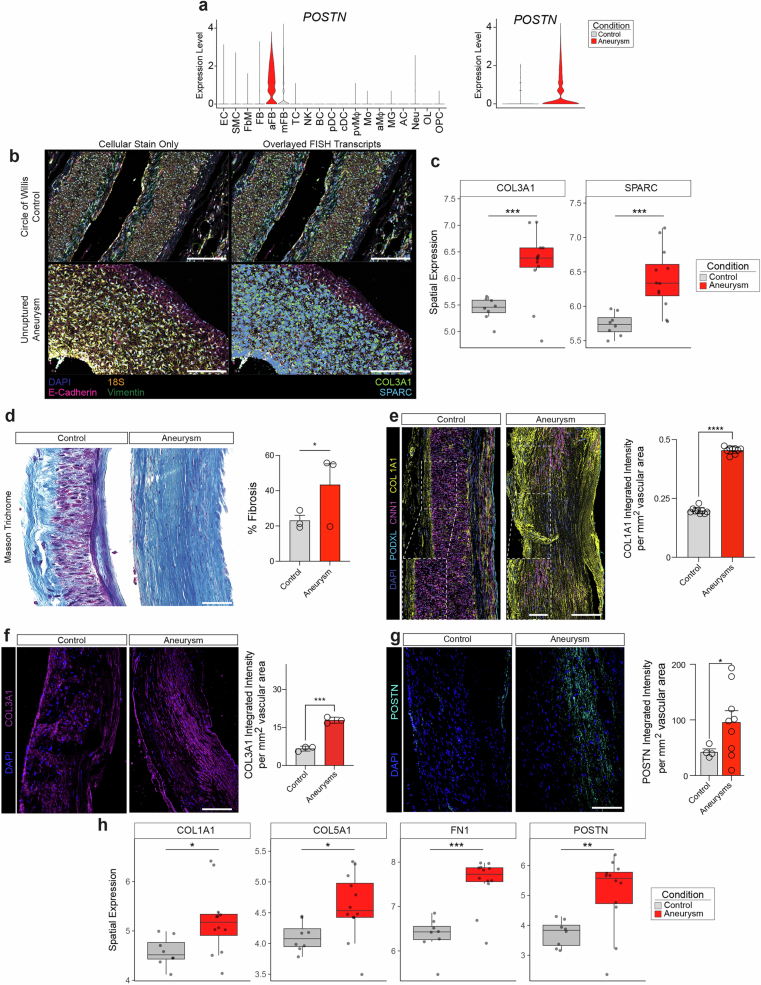
Fibrosis in brain aneurysms. **a**, Left: violin plot showing expression of *POSTN* across cell populations. Right: violin plot showing expression of *POSTN* by condition. EC, endothelial cell; SMC, smooth muscle cell; FbM, fibromyocyte; FB, fibroblast; aFB, activated fibroblast; mFB, myofibroblast; TC, T-cell; NK, natural killer cell; BC, B-cell; pDC, plasmacytoid dendritic cell; cDC, conventional dendritic cell; pvMϕ, perivascular macrophage; Mo, monocyte; aMϕ, *APC5*^*+*^ macrophage; MG, microglia; AC, astrocyte; Neu, neuron; OL, oligodendrocyte; OPC, oligodendrocyte precursor cell. **b**, Representative fluorescent *in situ* hybridization (FISH) analysis of *COL3A1* and *SPARC* transcripts on Circle of Willis controls (upper row) and unruptured aneurysm (lower row). Left: cellular fluorescent segmentation stain consisting of 18S (orange), E-cadherin (magenta), Vimentin (green). Right: merged image showing *COL3A1*^*+*^ (green) and *SPARC*^*+*^ (blue) fluorescent *in situ* hybridization (FISH) transcripts staining overlayed on cellular stain. Dot size scales with the number of overlapping transcripts detected. Scale bar, 200 µm. **c**, Box and whiskers plot of *COL3A1* (left) and *SPARC* (right) spatially determined gene expression levels. *COL3A1:* (FDR = 0.000850, ***), *SPARC* (FDR = 0.000553, ***). (n = 8 control sections, n = 12 unruptured brain aneurysm sections; two-sided *t*-test). Center lines indicate medians; box limits indicate the 25th and 75th percentiles; whiskers extend to the most extreme data points no more than 1.5 × IQR from the box; points beyond the whiskers are shown as outliers. **d**, Left: representative Masson’s Trichome histological stain showing extent of fibrosis in control middle cerebral artery and unruptured brain aneurysm. Scale bar, 100 µm. Blue, collagen-rich extracellular matrix. Purple, smooth muscle fibers. Right: integrated density quantifications of fibrosis between control middle cerebral arteries and brain aneurysms. (*P* = 0.0383, *, n = 3 control middle cerebral arteries, n = 3 aneurysms; two-sided *t-*test, mean ± s.e.m). **e**, Left: representative confocal microscopy analysis of *PODXL*^+^ endothelial cells (cyan), *CNN1*^+^ smooth muscle cells (magenta), and *COL1A1*^+^ extracellular matrix (yellow) in control middle cerebral arteries and brain aneurysms. Colocalization, white. Scale bar, 100 μm; Inset scale bar, 50 µm. Right: quantifications of *COL1A1*^*+*^ integrated density. (*P* = 7.36×10^−14^, ****, n = 9 control middle cerebral arteries, n = 9 aneurysms; two-sided *t*-test; mean ± s.e.m). **f**, Left: representative confocal microscopy analysis of COL3A1^+^ extracellular matrix (magenta) in control middle cerebral artery and brain aneurysm. Scale bar, 100 µm. Right: integrated density quantifications between control middle cerebral arteries and brain aneurysms. (*P* = 0.0003, ***, n = 3 control middle cerebral arteries, n = 3 aneurysms; two-sided *t-*test, mean ± s.e.m). **g**, Left: representative confocal microscopy analysis of *POSTN*^+^ immunostaining (cyan) in control middle cerebral artery and brain aneurysm. Right: integrated density quantifications between control middle cerebral arteries and brain aneurysms. (*P* = 0.035, *, n = 4 control middle cerebral arteries, n = 9 aneurysms; two-sided *t*-test; mean ± s.e.m). **h**, Box and whiskers plots of *COL1A1*, *COL5A1*, *FN1*, and *POSTN*. *COL1A1*: (FDR = 0.0120, *), *COL5A1*: (FDR = 0.0120, *), *FN1*: (FDR = 0.000136, ***), *POSTN*: (FDR = 0.00512, **). (n = 8 control sections, n = 12 unruptured brain aneurysm sections; two-sided *t*-test).

Environmental and genetic factors can contribute to the development of brain aneurysms through changes in the composition and gene expression of cerebrovascular cells^7,8^. To understand these changes at a cellular level, we first compared cell composition between control and aneurysm tissue samples within our cell atlas. Brain aneurysms showed significant depletion in specific vascular cell populations, particularly smooth muscle cells (Fig. 3a)^20,21^. We also observed reduced vascular smooth muscle cells in situ with cell-resolution spatial transcriptomics and immunostaining (Fig. 3b,c). By contrast, perivascular fibroblasts were more prevalent and were observed to replace much of the vascular wall in aneurysms (Figs. 2b and 3a,b,d). This was the result of a selective emergence of *POSTN*^*+*^ perivascular fibroblasts in brain aneurysms, which are infrequently detected in control cerebral arteries (Fig. 3a,b,d and Extended Data Fig. 4a). Using rigorously benchmarked bulk RNA-seq deconvolutions (Supplementary Fig. 1a–d), we similarly observed enrichment of *POSTN*^*+*^ perivascular fibroblasts in an expanded cohort of aneurysms widely distributed in the human cerebral circulation (*n* = 16 control cerebral arteries; *n* = 43 aneurysms) (Supplementary Fig. 1e,f and Supplementary Table 1)^7,22^. Spatial transcriptomics and quantitative immunostaining further confirmed a significantly increased abundance of *POSTN*^*+*^ perivascular fibroblasts in brain aneurysms (*P* < 0.05) and revealed that they are most prominently distributed in the tunica media, the normal cytoarchitectural location of vascular smooth muscle cells (Fig. 3b,d)^23^.

**Extended Data Fig. 5:**
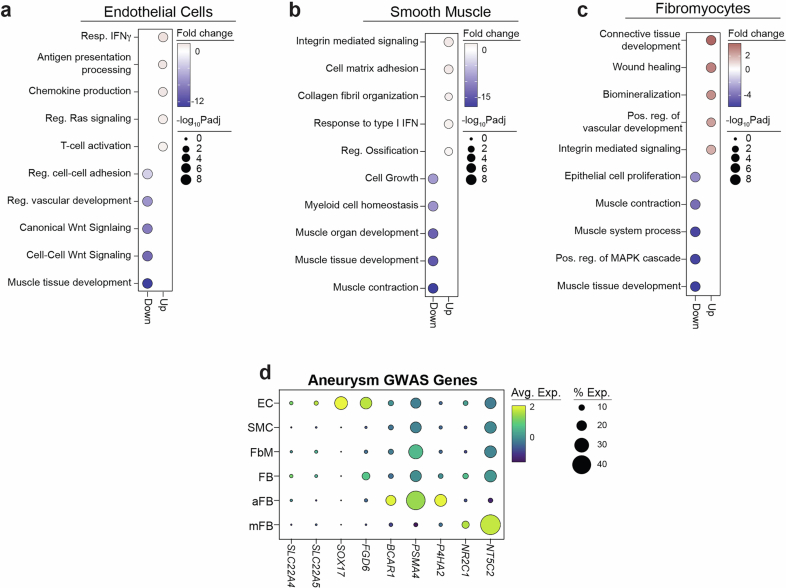
Diseased gene expression in vascular cells of brain aneurysms. **a-b**, Dot plot of enriched biological process in aneurysm arterial endothelial cells (**a**), smooth muscle cells (**b**), and fibromyocytes (**c**) (Bonferroni-corrected p-value < *0.05*, based on Single-Cell Pathway Analysis). Red, upregulated; blue, downregulated. **d**, Dot plot of cell-specific expression of individual brain aneurysm GWAS genes. Avg., average; Exp., expression; Blue, low expression; Yellow, high expression. EC, Endothelial Cell; SMC, Smooth Muscle Cell; FbM, Fibromyocyte; FB, Fibroblast; aFB, Activated Fibroblast; mFB, Myofibroblast.

Next, we profiled vascular cell-specific gene expression in controls and aneurysms from the cell atlas. We quantified the degree to which each vascular cell population is transcriptionally perturbed in aneurysms compared to controls by performing jackknife resampling on a transcriptome-wide analysis of differential expression (TRADE)^24^. This analysis provides a transcriptome impact score, a per-cell metric that compares each aneurysm cell to its most similar matching control cell and summarizes the global differential expression signal. The transcriptome impact score, therefore, provides an estimate of the perturbational impact of the aneurysmal pathology on cells compared to controls. Using this framework, perivascular fibroblasts, followed by smooth muscle cells, had the greatest transcriptional perturbation, supporting that perivascular cell populations were most affected in aneurysms (Fig. 3e). Perivascular fibroblasts upregulated profibrotic extracellular matrix genes, such as collagens, for example, *COL3A1* and *SPARC* (encodes osteonectin)^25,26^, which were validated in situ with spatial gene expression (Extended Data Fig. 4b, c). Fibrosis is defined cytoarchitecturally by the excessive production and deposition of extracellular matrix components^27^, and we further confirmed fibrosis in brain aneurysms with histology and in situ gene expression (Extended Data Fig. 4d–h). Cell-resolution pathway analyses also revealed concurrent downregulated expression of genes associated with multiple vessel-stabilizing pathways, including fibroblast-derived smooth muscle regulatory cascades, smooth muscle contractile proteins and growth, and barrier-promoting endothelial *Wnt* signaling. (Fig. 3f, Extended Data Fig. 5a–c and Supplementary Table 3). Aneurysmal vascular cells also showed higher levels of gene expression associated with baseline inflammation, such as leukocyte chemotaxis, antigen processing and presentation, and chemokine production (Fig. 3f, Extended Data Fig. 5a–c and Supplementary Table 3). Together, these data suggest widespread fibrosis and inflammation within the vascular wall and provide a cell type-specific molecular basis for vascular destabilization in brain aneurysms.

## Vascular cell contributors to brain aneurysm genetic risk

Heritable genetic variation is an important contributor to brain aneurysm formation^28,29^. Genome-wide association studies (GWAS) have identified candidate brain aneurysm risk loci and previously mapped several nominated genes to endothelial cells^28,29^. These studies, however, utilized non-aneurysmal cellular datasets that lacked representation of other vascular cells and were primarily generated from mice. We, therefore, investigated whether potential genetic contributions from other vascular cells may have been overlooked in humans.

We curated GWAS brain aneurysm risk genes^28,29^ and mapped their expression within our cell census (Extended Data Fig. 5d). Several brain aneurysm risk genes showed cell-specific patterns of expression, including two genes previously found to be expressed in endothelial cells, *SOX17* and *FDG*^30,31^ (Extended Data Fig. 5d). Of note, the expression of other brain aneurysm risk genes was enriched in *POSTN*^*+*^ perivascular fibroblasts, including *BCAR1, PSMA4* and *P4HA2* (Extended Data Fig. 5d), supporting a potential pathological role for these cells in brain aneurysms. To further prioritize aneurysm-associated cell populations from common GWAS risk loci, we performed two complementary genome-wide approaches: MAGMA^32^, which tests whether polygenic risk loci are enriched in and around genes highly expressed in pseudobulked expression profiles of each cell type, and scPagwas^33^, which aggregates polygenic risk loci by gene pathway utilization on a single-cell basis. Using summary statistics from a large-scale GWAS study (*n* = 10,754 aneurysms and *n* = 306,882 controls; Fig. 3g), both analyses identified significant associations of *POSTN*^*+*^ perivascular fibroblasts and smooth muscle cells with brain aneurysms (false discovery rate (FDR) < 0.05; Fig. 3g), further nominating these two perivascular cell types as being highly susceptible to the heritability of aneurysms. Top trait-relevant pathways identified in scPagwas included cellular senescence in smooth muscle cells (hsa04218; *P* < 0.001) and proimmune and fibrotic pathways, such as Toll-like receptor (hsa04620; *P* < 0.001) and TGFβ signaling (hsa04350; *P* < 0.001), in *POSTN*^*+*^ perivascular fibroblasts. Collectively, our data support contributions of multiple vascular cell types beyond endothelial cells, including smooth muscle cells and *POSTN*^*+*^ perivascular fibroblasts, to the heritable risk of brain aneurysms.

## Brain aneurysm fibroblast activation

**Fig. 4:**
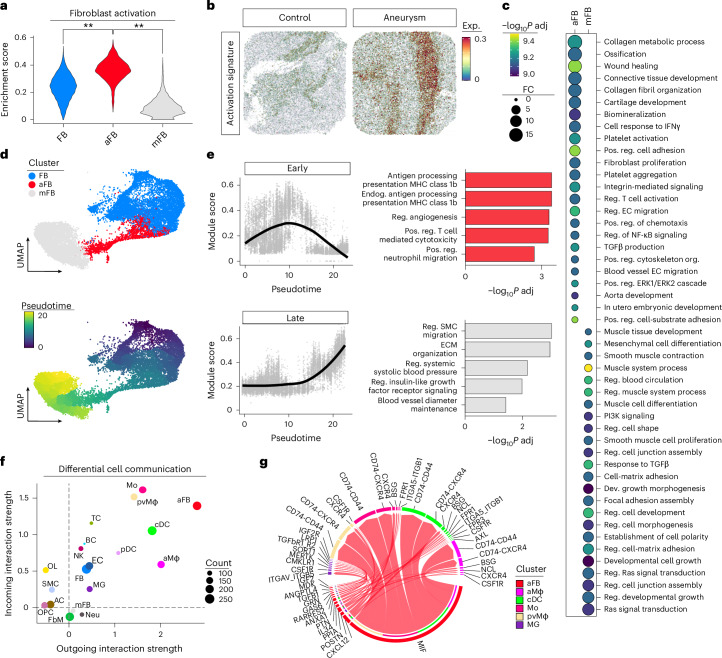
Perivascular fibroblast activation and diversity in brain aneurysms. **a**, Violin plot showing expression of fibroblast activation gene signature from Chaffin et al.^35^. **P < 0.01 (one-way analysis of variance (ANOVA) with Tukey’s multiple comparisons test). **b**, Spatial feature plot utilizing Slide-Seq’s transcriptome-wide spatial transcriptomics assay to show expression of fibroblast activation signature (top 20 genes ranked by limma-voom log_2_ fold change (FC) from Chaffin et al.^35^) in non-aneurysmal posterior cerebral artery (left) and unruptured brain aneurysm (right). **c**, Dot plot of enriched biological processes from aFB and mFB populations (Bonferroni-corrected *P* value < 0.05, based on single-cell pathway analysis). **d**, UMAP of 16,687 perivascular fibroblast transcriptomes colored by cell identity (top) or pseudotime (bottom). **e**, Left: scatter-plot showing early (top) or late (bottom) gene module expression along pseudotime activation axis. Right: enriched functional pathways of genes contained within early (top) and late (bottom) gene modules ordered by Benjamini–Hochberg adjusted *P* value (Fisher’s exact test). **f**, Scatter-plot showing differential outgoing (*x* axis) and incoming (*y* axis) interaction strength across cell populations in aneurysms compared to controls. Dot size represents number of cell-to-cell interactions in aneurysm (count). Population size parameter was turned off to remove artifacts introduced by uneven population distributions. **g**, Chord plot showing outgoing cell signaling pathways originating from activated perivascular fibroblasts to myeloid cells in aneurysms. Arrow thickness is proportional to interaction strength. Endog., endogenous; org., organization; Dev., developmental.

Fibroblast activation occurs in other human cardiovascular diseases, most notably heart disease^34,35^. *POSTN* is an established fibroblast activation marker^34,35^ so we explored whether fibroblast activation contributes to the emergence of *POSTN*^*+*^ perivascular fibroblasts in brain aneurysms. Using an established fibroblast activation transcriptional signature^35^, we found enriched expression in *POSTN*^*+*^ perivascular fibroblasts but not other perivascular fibroblast populations in the cell atlas (Fig. 4a and Supplementary Table 4). Genome-wide spatial transcriptomics further confirmed the in situ expression of this fibroblast activation signature (Fig. 4b and Supplementary Table 4). A comparison of enriched biological processes between the fibroblast populations identified in the cell atlas suggested a functional specialization of the *POSTN*^*+*^ perivascular fibroblasts. Specifically, *POSTN*^*+*^ perivascular fibroblasts were enriched in immune and wound response pathways (Fig. 4c). Notably, there was also enrichment in terms that suggest a role in vascular calcification, a phenomenon observed in many brain aneurysms (Fig. 4c)^36^. Conversely, we also identified a distinct *LAMB1*^+^ fibroblast population enriched for smooth muscle and contractile gene programs (Fig. 4c), consistent with a myofibroblast-like state typically observed during later stages of wound healing and fibrotic remodeling^37,38^.

**Extended Data Fig. 6:**
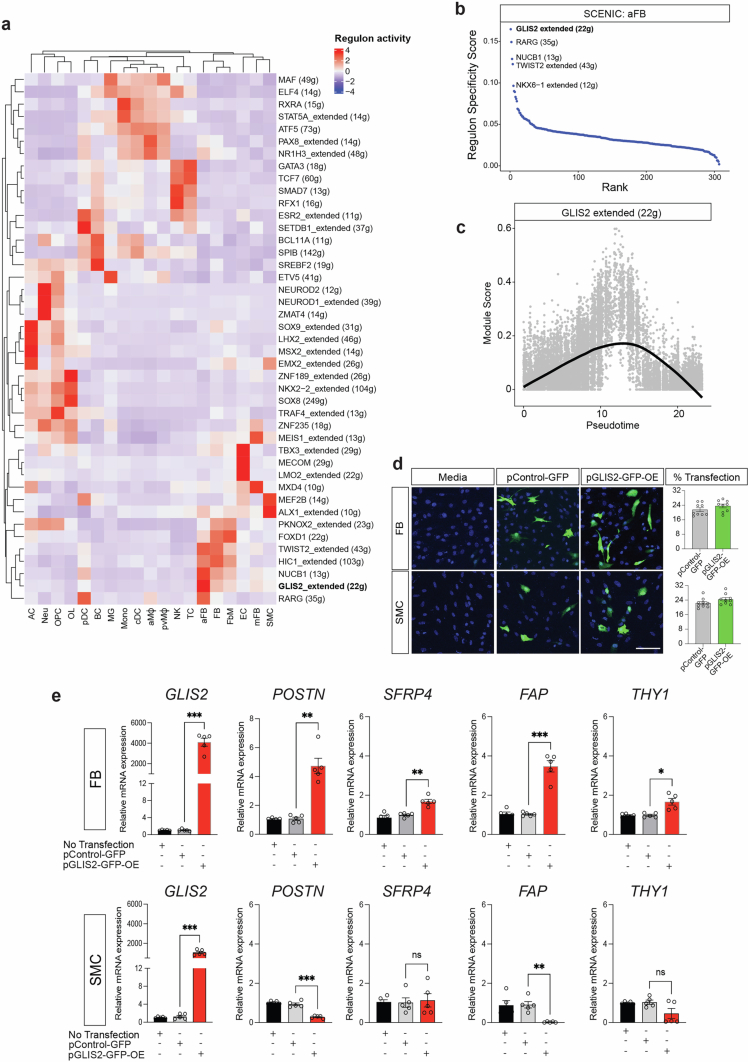
Genetic regulators of perivascular fibroblast activation. **a**, Heatmap showing regulon activity across cell types grouped by hierarchical clustering. Red, high activity; blue, low activity. *x*-axis, cell-type; *y*-axis, regulon. The number of genes is listed in parentheses to the right of the regulon name. Bold, top nominated regulon activated fibroblasts identified by Single-Cell rEgulatory Network Inference and Clustering (SCENIC). EC, endothelial cell; SMC, smooth muscle cell; FbM, fibromyocyte; FB, fibroblast; aFB, activated fibroblast; mFB, myofibroblast; TC, T-cell; NK, natural killer cell; BC, B-cell; pDC, plasmacytoid dendritic cell; cDC, conventional dendritic cell; pvMϕ, perivascular macrophage; Mo, monocyte; aMϕ, *APC5*^*+*^ macrophage; MG, microglia; AC, astrocyte; Neu, neuron; OL, oligodendrocyte; OPC, oligodendrocyte precursor cell. **b**, Rank plot of each regulon by the regulon specificity score in aFB. Names of the top five regulons shown with the number of included genes in parenthesis to the right. **c**, Scatter-plot showing *GLIS2* regulon gene module along fibroblast pseudotime activation axis. **d**, Left: representative epifluorescent microscopy images of cultured primary human adventitial fibroblasts (top) and smooth muscle cells (bottom). pControl-GFP, Green fluorescent protein (GFP)-tagged control plasmid; pGLIS2-GFP-OE, GFP-tagged *GLIS2*-overexpressing plasmid. DAPI (blue) shows cell nuclei. Scale bar, 100 µm. Right: Bar plot of percent transfection efficiency in pControl-GFP (gray) and pGLIS2-GFP-OE (green). Mean ± s.e.m (n = 9 samples). FB, Fibroblast; SMC, Smooth Muscle Cell. **e**, Bar plot showing quantitative polymerase chain reaction (qPCR) analysis of *GLIS2* and fibroblast activation genes (*POSTN*, *SRP4*, *FAP*, *THY1*) in primary human adventitial fibroblasts (top) and smooth muscle cell (bottom) lines following *GLIS2* overexpression. Mean ± s.e.m. FB: *GLIS2* (*P* = 0.0005, ***), *POSTN* (*P* = 0.0019, **), *SFRP4* (*P* = 0.0017, **), *FAP* (*P* = 0.0009, ***), *THY1* (*P* = 0.0129, *). SMC: *GLIS2* (*P* = 0.0008, ***), *POSTN* (*P* = 0.0002, ***), *SFRP4* (*P* = 0.7859, ns), *FAP* (*P* = 0.0053, **), *THY1* (*P* = 0.0869, ns). One-way ANOVA with Tukey’s multiple comparisons test (n = 5 samples).

To understand further the transcriptional programs underlying perivascular fibroblast activation in brain aneurysms, we ordered the three fibroblast cell states identified in the cell atlas along a one-dimensional pseudotime axis (Fig. 4d)^39^. Unsupervised computation of genes that statistically varied along the pseudotime trajectory supported a transcriptional continuum between quiescent and activated perivascular fibroblasts. Early in this trajectory, we identified the upregulated expression of a gene module enriched in genes associated with immune responses, such as *SFRP2* and *FAP*, which have been implicated in pathogenic fibroblast transitions in other immune-mediated diseases, such as systemic sclerosis and rheumatoid arthritis (Fig. 4e and Supplementary Table 5)^38,40^. By contrast, a later trajectory gene module was enriched in genes associated with myogenic and contractility genes (Fig. 4e), consistent with the acquisition of a wound-healing myofibroblast-like state. To identify the regulators of perivascular fibroblast activation in brain aneurysms, we applied SCENIC^41^, which infers gene regulatory networks and transcription factors, to our cell atlas and identified transcription factors selectively enriched in *POSTN*^*+*^ perivascular fibroblasts. The zinc finger protein *GLIS2* emerged as the top enriched transcription factor in *POSTN*^*+*^ perivascular fibroblasts, which is also implicated in autosomal dominant polycystic kidney disease^42^, a condition with elevated risk for brain aneurysms^43–48^ (Extended Data Fig. 6a–c). Overexpression of *GLIS2* in primary brain perivascular fibroblasts, but not in smooth muscle cells, recapitulated *POSTN*^*+*^ perivascular fibroblast activation gene signatures with increased expression of *POSTN*, *FAP*, *SFRP4* and *THY1*^38,40^ (Extended Data Fig. 6d, e and Supplementary Table 6). Thus, perivascular fibroblast activation is associated with pathologic cell transitions that are transcriptionally similar to other human cardiovascular and immune-mediated diseases.

**Extended Data Fig. 7:**
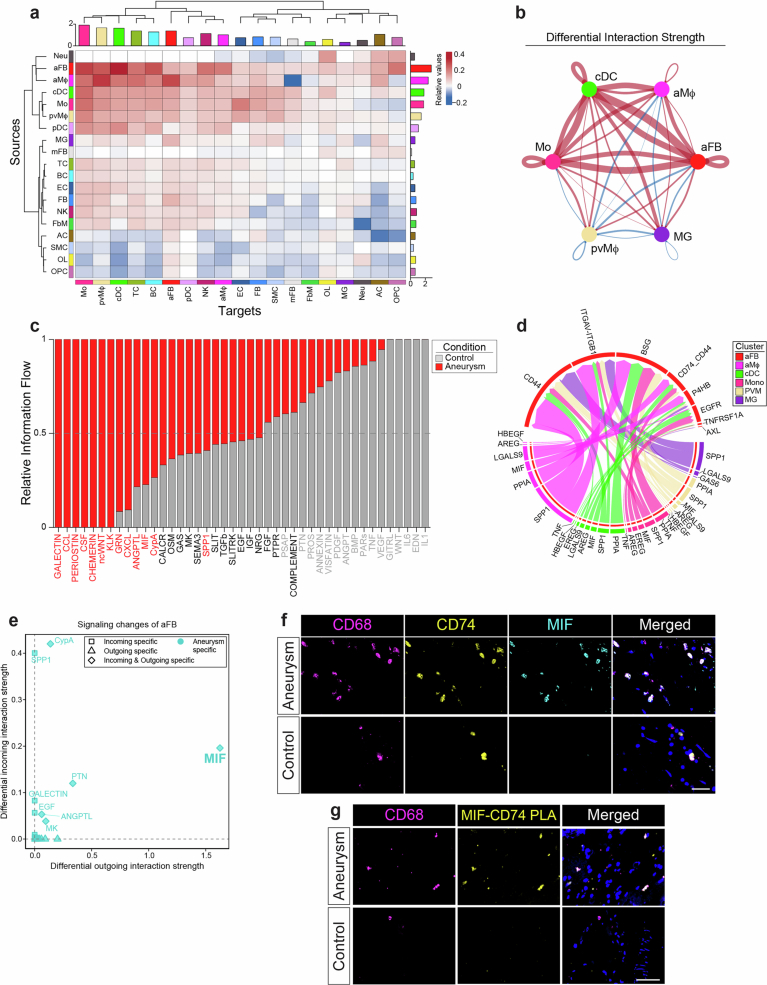
Dysregulated cell communication networks in brain aneurysms. **a**, Heatmap with hierarchical clustering showing cell-to-cell differential interaction strength in brain aneurysms when compared to control cerebrovasculature. *y*-axis, source; *x*-axis, target. Red, increased interaction strength in aneurysms; blue, decreased interaction strength in aneurysms. EC, endothelial cell; SMC, smooth muscle cell; FbM, fibromyocyte; FB, fibroblast; aFB, activated fibroblast; mFB, myofibroblast; TC, T-cell; NK, natural killer cell; BC, B-cell; pDC, plasmacytoid dendritic cell; cDC, conventional dendritic cell; pvMϕ, perivascular macrophage; Mo, monocyte; aMϕ, *APC5*^*+*^ macrophage; MG, microglia; AC, astrocyte; Neu, neuron; OL, oligodendrocyte; OPC, oligodendrocyte precursor cell. **b**, Circle plot of differential interaction strength between activated perivascular fibroblasts (aFB) and myeloid cell populations in aneurysms. Red, increased interaction strength in aneurysms; blue, decreased interaction strength in aneurysms. Line thickness proportional to interaction strength. **c**, Cell-to-cell communication pathways across cell populations ranked on their differences of overall information flow within inferred networks in aneurysms (red) and control (gray). Red text, pathways statistically over-represented in brain aneurysms; gray text, pathways statistically over-represented in controls; black text, not significant. **d**, Chord plot showing outgoing cell communication pathways originating from myeloid cells to activated perivascular fibroblasts in aneurysms. Arrow thickness is proportional to interaction strength. **e**, Scatter-plot of pathways ranked by differential incoming (*y*-axis) and outgoing (*x*-axis) interaction strength in aFB in brain aneurysms relative to controls. Light blue, aneurysm-specific. Bold, top-nominated outgoing pathway emerging from aFB. **f**, Representative confocal microscopy analysis of immunostaining *CD68*^*+*^ (magenta) macrophages showing colocalization with CD74 receptor (yellow) and MIF ligand (cyan) in unruptured brain aneurysm (top) and control middle cerebral artery (bottom). DAPI (blue) labels cell nuclei. Colocalization, white. Scale bar, 50 µm. **g**, Representative confocal microscopy analysis of CD68^+^ macrophages (magenta) and MIF–CD74 proximity interaction (yellow) in unruptured brain aneurysm (top) and control middle cerebral artery (bottom). DAPI (blue) labels cell nuclei. Colocalization, white. Scale bar, 50 µm.

We next investigated whether activated *POSTN*^*+*^ perivascular fibroblasts have heightened crosstalk with immune cells. Using reciprocal ligand–receptor interactions^49^, we unbiasedly queried cell-to-cell communication pathways in controls and brain aneurysms in our cell atlas^49^. Comparison of the assembled interactomes nominated *POSTN*^*+*^ perivascular fibroblasts as the strongest contributor to cell communications in brain aneurysms (Fig. 4f). Differential comparison in pairwise signaling strengths between cell types in our cell atlas suggested increased crosstalk between *POSTN*^*+*^ perivascular fibroblasts and select myeloid cells (Extended Data Fig. 7a, b). Many over-represented pathways in this crosstalk between *POSTN*^*+*^ perivascular fibroblasts and myeloid cells were proimmune signaling pathways, for example, colony-stimulating factor, macrophage inhibitory factor (MIF) and CC or CX chemokine ligand–receptor signaling, or established vascular destabilizing cascades, for example, cyclophilin A (CypA) or secreted phosphoprotein 1 (SPP1) (Fig. 4g and Extended Data Fig. 7c, d)^9,50,51^. Cell-to-cell communication analysis identified MIF as the most dysregulated outgoing ligand from *POSTN*^*+*^ perivascular fibroblasts in brain aneurysms (Extended Data Fig. 7e). MIF signals via the receptor CD74 on macrophages^52^. Consistent with this, immunostaining and a MIF–CD74 proximity ligation assay (PLA) demonstrated increased MIF–CD74 colocalization and ligand–receptor complex formation in *CD68*^+^ macrophages in brain aneurysms compared to controls (Extended Data Fig. 7f, g). Together, these data support heightened MIF–CD74 signaling and a previously overlooked myeloid–perivascular fibroblast communication axis in brain aneurysms.

## Inflammation and a specialized myeloid cell in brain aneurysms

**Extended Data Fig. 8:**
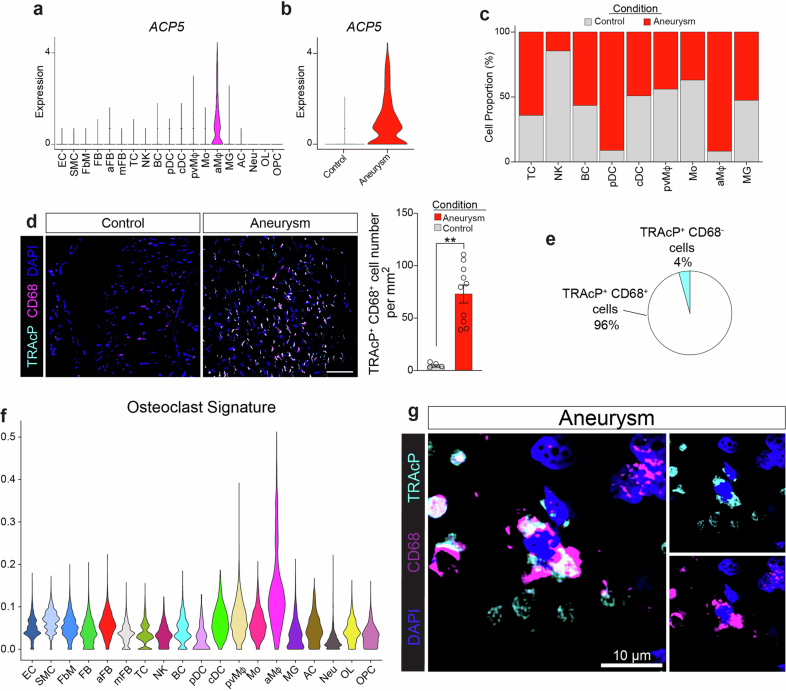
Activated macrophages in brain aneurysms and controls. **a**, Violin plot of *ACP5* across cell populations. EC, endothelial cell; SMC, smooth muscle cell; FbM, fibromyocyte; FB, fibroblast; aFB, activated fibroblast; mFB, myofibroblast; AC, astrocyte; Neu, neuron; OL, oligodendrocyte; OPC, oligodendrocyte precursor cell. **b**, Violin plot of *ACP5* expression across conditions. Gray, control; Red, aneurysm. **c**, Bar graph of cell proportion by condition from control cerebrovasculature (gray) and brain aneurysms (red) highlighting enrichment of *APC5*^*+*^ macrophages in brain aneurysms. **d**, Left: representative confocal microscopy analysis and quantification of TRAcP^+^ (cyan, encoded by *ACP5*) and CD68^+^ (magenta) macrophages in control middle cerebral artery and brain aneurysms. Colocalization, white. DAPI (blue) shows cell nuclei. Scale bar, 50 μm. Right: Bar graph of quantification of TRAcP^+^ CD68^+^ cells in control middle cerebral arteries (n = 4 donors) and aneurysms (n = 10 donors). (*P* = 0.002, **, two-sided *t*-test, mean ± s.e.m). **e**, Pie chart of specificity of TRAcP immunostaining in brain aneurysms. White pie, percentage of TRAcP^+^ cells that are CD68^+^; Cyan pie, percentage of TRAcP^+^ cells that are CD68^−^. **f**, Violin plot showing expression of osteoclast gene signature (defined by Tsukasaki et al. 2020) across cell types. EC, endothelial cell; SMC, smooth muscle cell; FbM, fibromyocyte; FB, fibroblast; aFB, activated fibroblast; mFB, myofibroblast; TC, T-cell; NK, natural killer cell; BC, B-cell; pDC, plasmacytoid dendritic cell; cDC, conventional dendritic cell; pvMϕ, perivascular macrophage; Mo, monocyte; aMϕ, *APC5*^*+*^ macrophage; MG, microglia; AC, astrocyte; Neu, neuron; OL, oligodendrocyte; OPC, oligodendrocyte precursor cell. **g**, Representative high-magnification confocal microscopy analysis of a TRAcP^+^ (cyan) and CD68^+^ (magenta) macrophage in brain aneurysm tissue. Scale bar, 10 µm.

Myeloid-driven inflammation plays a crucial role in the development of brain aneurysms^53,54^. Using our human aneurysm cell atlas, we classified immune cell populations based on canonical gene marker expression (Supplementary Fig. 2a). Overall, we identified nine immune cell populations (Fig. 1c, d and Supplementary Fig. 2a). Five immune cell populations were composed of myeloid cells, including perivascular macrophages (pvMϕs), conventional dendritic cells (cDCs) and monocytes (Mo). Marker gene profiles for each canonical immune subset aligned with their established roles in neuroimmunology, for example, microglial modulation of synaptic function and dendritic cell major histocompatibility complex class II-mediated antigen presentation^55–57^ (Supplementary Fig. 2b). Consistent with previous reports^7,8^, cell-resolution pathway enrichment analyses showed broad activation of proinflammatory and effector programs, for example, chemotaxis, antigen processing and lymphocyte-mediated cytotoxicity, with concurrent reductions in vascular-stabilizing pathways, for example, wound healing, cell adhesion and barrier integrity, across immune cell populations in brain aneurysms (Supplementary Fig. 3). Of note, we identified a unique myeloid cell population that highly coexpressed *ACP5* (encodes tartrate resistance acid phosphatase 5 (TRAcP)) and the macrophage (Mϕ) marker *CD68* (Extended Data Fig. 8a, b). *ACP5*^*+*^ Mϕs have not been previously detected in the human cerebrovasculature^9,10^, and these cells were much more abundant in brain aneurysms (Extended Data Fig. 8c). Immunostaining confirmed the presence of TRAcP^+^ Mϕs in aneurysms, which were rarely detected in controls in situ (Extended Data Fig. 8d, e). Notably, TRAcP^+^ Mϕs bear transcriptional resemblance to osteoclasts due to the expression of *ACP5* and other markers^58^, but high-resolution immunostaining revealed they were not multinucleated^59^ (Extended Data Fig. 8f, g and Supplementary Table 4). Thus, we identified an *ACP5*^*+*^ Mϕ population previously unrecognized in human brain aneurysms or other cerebrovascular diseases.

**Extended Data Fig. 9:**
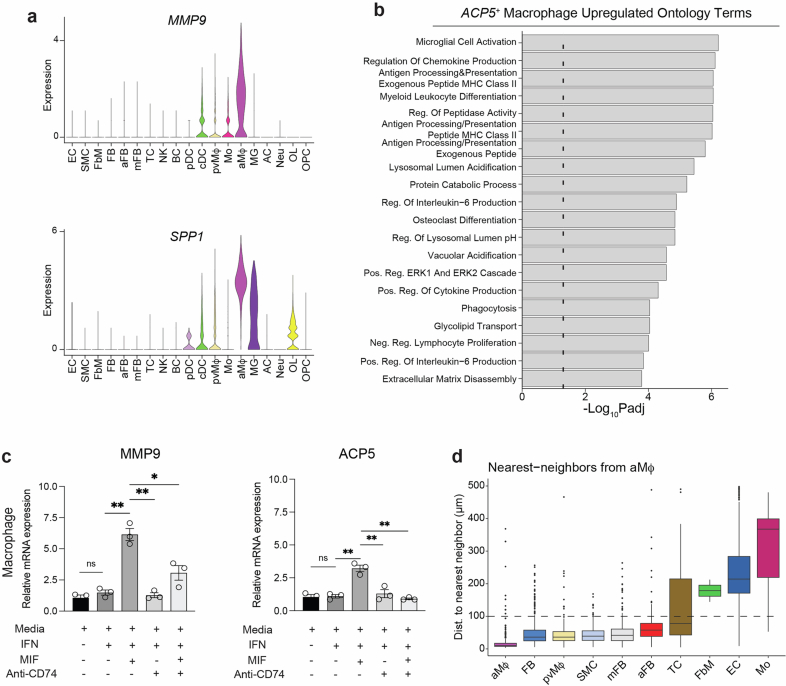
Function and spatial context of activated macrophages in brain aneurysms and controls. **a**, Violin plot of the established vascular destabilizing genes *MMP9* and *SPP1* across cell populations. EC, endothelial cell; SMC, smooth muscle cell; FbM, fibromyocyte; FB, fibroblast; aFB, activated fibroblast; mFB, myofibroblast; TC, T-cell; NK, natural killer cell; BC, B-cell; pDC, plasmacytoid dendritic cell; cDC, conventional dendritic cell; pvMϕ, perivascular macrophage; Mo, monocyte; aMϕ, *APC5*^*+*^ macrophage; MG, microglia; AC, astrocyte; Neu, neuron; OL, oligodendrocyte; OPC, oligodendrocyte precursor cell. **b**, Enriched biological processes from marker genes in *ACP5*^*+*^ macrophages ordered by statistical significance Benjamini-Hochberg adjusted *P* < 0.05; dashed line, Benjamini-Hochberg adjusted *P* cutoff of 0.05. **c**, Bar graph showing quantitative polymerase chain reaction (qPCR) analysis of *MMP9* (left) and *ACP5* (right) expression in primary human macrophages treated with interferon (IFN), macrophage inhibitory factor (MIF), and CD74 neutralizing antibody (anti-CD74). Mean ± s.e.m. MMP9: Media vs IFN (*P* = 0.2479, ns); IFN vs IFN + MIF (*P* = 0.0047, **); IFN + MIF vs IFN+anti-CD74 (*P* = 0.0046, **); IFN + MIF vs IFN + MIF+anti-CD74 (*P* = 0.017, *). ACP5: Media vs IFN (*P* = 0.8114, ns); IFN vs IFN + MIF (*P* = 0.0052, **); IFN + MIF vs IFN+anti-CD74 (*P* = 0.009, **); IFN + MIF vs IFN + MIF+anti-CD74 (*P* = 0.009, **). One-way ANOVA with Tukey’s multiple comparisons test (n = 3 samples). **d**. Boxplot of the distance (µm) from each aMϕ to its nearest neighbor of each listed cell type (n = 770 aMϕ). Dashed line marks 100 µm. Center lines indicate medians; box limits indicate the 25th and 75th percentiles; whiskers extend to the most extreme data points no more than 1.5 × IQR from the box; points beyond the whiskers are shown as outliers.

Immune cells degrade ECM proteins or promote vascular cell loss, contributing to the growth and/or rupture of brain aneurysms^60,61^. We therefore wondered whether *ACP5*^*+*^ Mϕs contribute to these aneurysm-destabilizing cell programs. Established ECM degradation gene products or inducers of smooth muscle cell death, for example, *MMP9* and *SPP1*, were among the top five *ACP5*^*+*^ Mϕ cell marker genes and more highly expressed in this cell population than other immune cells (Extended Data Fig. 9a)^9,62^. Pathway enrichment analysis of marker genes further supported the functional specialization of *ACP5*^*+*^ Mϕs, including enrichment of immune activation, phagocytic and ECM destabilizing cascades (Extended Data Fig. 9b). Based on our cell-to-cell communication analyses (Fig. 4g and Extended Data Fig. 7), we hypothesized that MIF secreted from *POSTN*^+^ perivascular fibroblasts may promote this macrophage cell state. In primary human macrophages, recombinant MIF significantly increased *ACP5* and *MMP9* expression (*P* < 0.01), and this induction was attenuated by a CD74-blocking antibody in vitro (Extended Data Fig. 9c). Furthermore, nearest neighborhood analysis on our spatial atlas revealed that the median distance from *ACP5*^*+*^ Mϕs to *POSTN*^*+*^ perivascular fibroblasts are within the cellular distances expected for MIF paracrine signaling^63,64^ (Extended Data Fig. 9d), suggesting that this proposed communication axis between *ACP5*^*+*^ Mϕs to *POSTN*^*+*^ perivascular fibroblasts are spatially supported. Together, these data suggest that *ACP5*^*+*^ Mϕs respond to MIF signaling and may remodel the perivascular environment and promote inflammation in human brain aneurysms.

## Aneurysm rupture and subarachnoid hemorrhage

For some brain aneurysms, destabilizing cell programs progress to make the aneurysm fragile and eventually rupture, which results in catastrophic bleeding into the subarachnoid space, known as aneurysmal subarachnoid hemorrhage (aSAH)^65^. Death occurs in >50% of patients^66^ and aSAH is an important contributor to sudden death in adults^66,67^. Thus, understanding the cellular mechanisms that contribute to aneurysm rupture is of high medical importance.

**Fig. 5:**
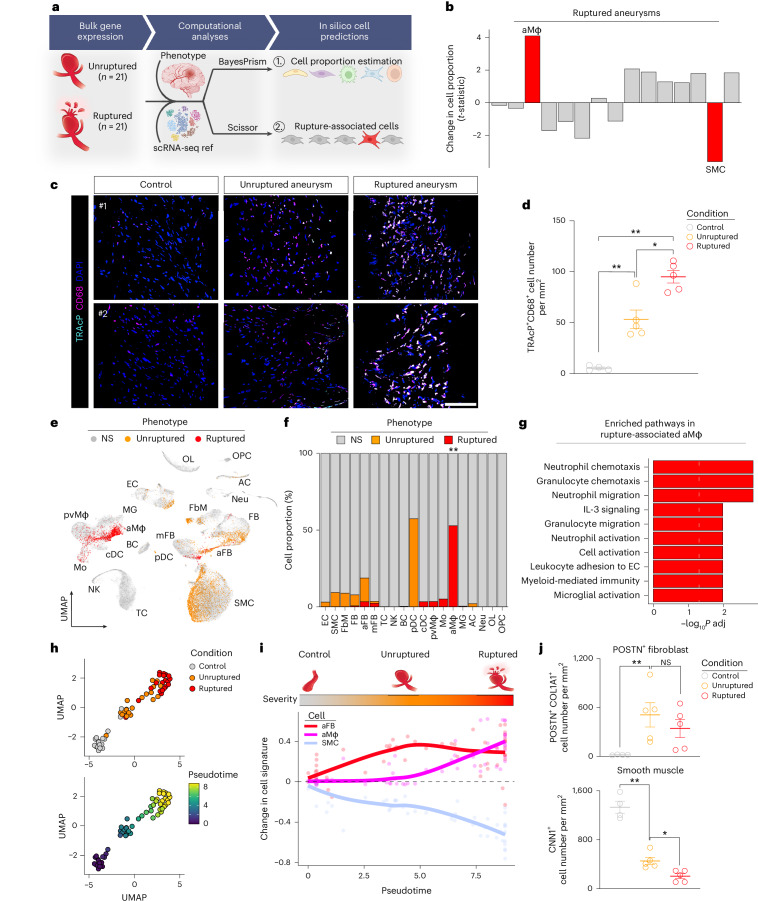
Cellular changes associated with aneurysm rupture and disease progression. **a**, Schematic demonstrating our integrated computational approach to identify cell-specific alterations in ruptured aneurysm. Bulk RNA-seq deconvolution performed on unruptured and ruptured aneurysms (unruptured, *n* = 21 donors; ruptured, *n* = 21 donors). **b**, Bar graph of change in cell population *t-*statistic in bulk sequenced ruptured aneurysms following cell deconvolution analysis. Red denotes statistical significance (FDR < 0.05 based on two-sided *t*-test, Benjamini–Hochberg correction). **c**, Representative confocal microscopy immunostaining of TRAcP^+^ (cyan, encoded by *ACP5*) and CD68^+^ (magenta) macrophages in a control middle cerebral artery, unruptured aneurysm and ruptured aneurysm. Colocalization, white. 4,6-Diamidino-2-phenylindole (DAPI) shows cell nuclei. Two representative examples are provided. Scale bar, 50 μm. **d**, Quantification of TRAcP^+^CD68^+^ macrophages in control middle cerebral arteries (*n* = 4 donors), unruptured aneurysms (*n* = 5 donors), and ruptured aneurysms (*n* = 5 donors). Control versus unruptured aneurysm (*P* = 0.0043**); unruptured versus ruptured aneurysm (*P* = 0.0317*); and control versus ruptured aneurysm (*P* = 0.0043**). One-way ANOVA with Tukey’s multiple comparisons test, mean ± s.e.m. **e**, UMAP of Scissor-selected cells utilizing phenotypic signatures between ruptured and unruptured aneurysms from bulk RNA sequencing (Kleinloog cohort)^7^. The red-colored cells are positively associated with aneurysm rupture (rupture-associated, *n* = 1,677) and orange-colored cells are negatively associated with aneurysm rupture (unruptured-associated, *n* = 4,681). NS, nonselected cells (gray). Differential gene expression signature was obtained from a comparison between ruptured and unruptured aneurysms and mapped onto unruptured aneurysm single-cell sequencing data. **f**, Bar plot showing the distribution of Scissor-selected rupture-associated cells (red) and non-rupture-associated cells (orange) across cerebrovascular and immune cell populations. NS, nonselected cells (gray). **Benjamini–Hochberg adjusted *P* < 0.01. Scissor categorization uses binomial logistic regression model. **g**, Bar graph of enriched biological processes in rupture-associated *ACP5*^*+*^ macrophages selected by Scissor compared with nonselected *ACP5*^*+*^ macrophages ordered by Benjamini–Hochberg adjusted *P* value (Fisher’s exact test). Differentially expressed genes used for enrichment analysis were calculated using a pseudobulked DESeq2 model. **h**, Top: UMAP projection showing unsupervised localization of identified samples by diagnosis and severity. Each point represents one sample. Gray, control cortical artery sample (*n* = 16 donors). Orange, unruptured aneurysm sample (*n* = 21 donors). Red, ruptured aneurysm sample (*n* = 21 donors). Bottom: UMAP projection of bulk transcriptomes ordered by unsupervised disease severity pseudotime analysis. **i**, Expression of cell signature scores as a function of disease severity pseudotime. Red, aFB; magenta, aMϕ; light blue, SMC. **j**, Top: quantification of immunostaining for POSTN^+^ COL1A1^+^ fibroblasts in control middle cerebral arteries (gray, *n* = 4 donors), unruptured aneurysms (orange, *n* = 5 donors), and ruptured aneurysms (red, *n* = 5 donors). Bottom: quantification of immunostaining for CNN1^+^ smooth muscle cells in control middle cerebral arteries (gray, *n* = 4 donors), unruptured aneurysms (orange, *n* = 5 donors) and ruptured aneurysms (red, *n* = 5 donors). Fibroblast: control versus unruptured aneurysm (*P* = 0.0079**), unruptured versus ruptured aneurysm (NS, not significant). Smooth muscle: control versus unruptured aneurysm (*P* = 0.0079**), unruptured versus ruptured aneurysm (*P* = 0.0159*). One-way ANOVA with Tukey’s multiple comparisons test, mean ± s.e.m. Panels created in BioRender: **a**, Winkler, E. https://BioRender.com/y1ghea1 (2026); **i**, Winkler, E. https://BioRender.com/0ini4zb (2026).

To explore the cellular pathology associated with aneurysm rupture, we leveraged our cell atlas to identify deleterious cell populations associated with aneurysm rupture. Using bulk RNA-seq deconvolution analysis in a previously reported cohort of unruptured (*n* = 21) and ruptured aneurysms (*n* = 21)^7^ (henceforth called the ‘Kleinloog’ cohort), we found statistically significant enrichment of *ACP5*^*+*^ Mϕs and depletion of smooth muscle cells in ruptured aneurysms (*P* < 0.01; Fig. 5a, b). As orthogonal validation, we immunostained an independent cohort of non-aneurysmal middle cerebral arteries (*n* = 4 donors) and unruptured (*n* = 5 donors) and ruptured (*n* = 5 donors) aneurysms to quantify the number of infiltrating TRAcP^+^ cells (TRAcP is encoded by *ACP5*) (Fig. 5c, d). Consistent with our in silico predictions, we found a statistically significant increase in the number of TRAcP^+^ cells with disease progression, and TRAcP^+^ Mϕs were most abundant in ruptured aneurysms (*P* < 0.01) (Fig. 5d). Thus, infiltration of *ACP5*^*+*^ Mϕs becomes more prevalent in ruptured brain aneurysms.

Next, we applied a computational approach called Scissor to integrate bulk RNA-seq phenotype data with our single-cell transcriptomic data to nominate cell states most strongly associated with aneurysm rupture (Methods)^68^. Scissor uses phenotype information from bulk RNA-seq data and assesses transcriptional similarity across cells from a single-cell reference to identify phenotype-associated cells, without requiring differential gene expression analysis as an intermediary step^68^. Thus, a rupture-associated transcriptional signature was calculated from the Kleinloog et al.^7^ bulk RNA-seq data (ruptured versus unruptured aneurysms) and then mapped onto our single-cell sequencing data derived exclusively from unruptured brain aneurysms. Of the 100,409 cells analyzed, the Scissor algorithm identified 1,677 cells that were positively correlated with aneurysm rupture and 4,681 cells that were negatively correlated with aneurysm rupture, hereafter referred to as rupture-associated and unruptured-associated cells, respectively. As expected, rupture-associated cells were frequently identified in brain aneurysms with high statistical confidence (hypergeometric test, *P* < 0.01). Most rupture-associated cells were identified in myeloid cell populations, especially *ACP5*^*+*^ Mϕs (*P* < 0.01; Fig. 5e, f). By contrast, rupture-associated cells were rarely detected in vascular cells and were limited to perivascular fibroblast populations (Fig. 5e, f). Subsequent differential gene expression analysis comparing rupture-associated and nonselected *ACP5*^+^ Mϕs revealed that rupture-associated *ACP5*^+^ Mϕs were functionally enriched for proinflammatory signaling programs, including chemokine and cytokine signaling and macrophage activation (Fig. 5g). Together, these data highlight a potential role for *ACP5*^*+*^ Mϕs in aneurysm rupture. However, future investigations are needed to better delineate whether these findings are a cause or consequence of aneurysm rupture.

## Natural history of cell alterations in brain aneurysms

**Extended Data Fig. 10:**
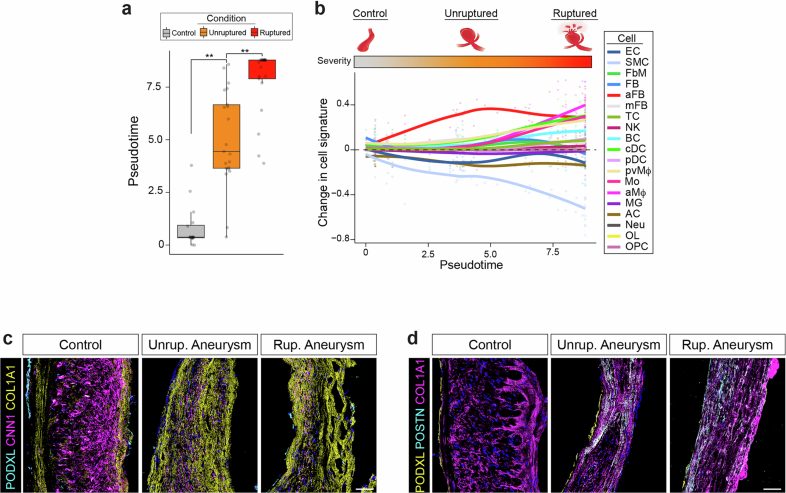
Cellular alterations with aneurysm progression. **a**, Box plot of disease severity pseudotime in bulk sequenced samples by condition. Gray, non-aneurysmal control (n = 16 donors); orange; unruptured aneurysm (n = 21 donors); red, ruptured aneurysm (n = 21 donors). Control vs Unruptured Aneurysm (*P* = 0, **), Unruptured vs Ruptured Aneurysm (*P* = 7.3×10^−6^, **). One-way ANOVA with Tukey’s multiple comparisons test. Center lines indicate medians; box limits indicate the 25th and 75th percentiles; whiskers extend to the most extreme data points no more than 1.5 × IQR from the box; points beyond the whiskers are shown as outliers. **b**, Expression of cell signature scores as function of disease severity pseudotime for all cell populations. EC, endothelial cell; SMC, smooth muscle cell; FbM, fibromyocyte; FB, fibroblast; aFB, activated fibroblast; mFB, myofibroblast; TC, T-cell; NK, natural killer cell; BC, B-cell; pDC, plasmacytoid dendritic cell; cDC, conventional dendritic cell; pvMϕ, perivascular macrophage; Mo, monocyte; aMϕ, *APC5*^*+*^ macrophage; MG, microglia; AC, astrocyte; Neu, neuron; OL, oligodendrocyte; OPC, oligodendrocyte precursor cell. Created in BioRender. Winkler, E. (2026) https://BioRender.com/0ini4zb. **c**, Representative confocal microscopy analysis of PODXL^+^ endothelial cells (yellow), POSTN^+^ perivascular fibroblasts (cyan), and COL1A1^+^ perivascular fibroblasts (magenta) in control middle cerebral arteries and brain aneurysms. Colocalization, white. Scale bar, 50 μm. **d**, Representative confocal microscopy analysis of PODXL^+^ endothelial cells (cyan), CNN1^+^ smooth muscle cells (magenta), and COL1A1^+^ perivascular fibroblasts (yellow) in control middle cerebral artery or brain aneurysm. Multiple representative examples shown. Scale bar, 50 μm.

Human brain aneurysms undergo multiple phases over their lifespan, for example, stable, smooth-walled aneurysms versus irregular, enlarging aneurysms with risks of rupture^60,61,69^. We therefore investigated whether differences in the aneurysm-associated cells found here, for example, *POSTN*^*+*^ perivascular fibroblasts and *ACP5*^*+*^ Mϕs, occur during aneurysm progression. We performed a pseudotime analysis on the Kleinloog cohort of bulk transcriptomes to order samples along a molecularly defined continuum of disease severity (*n* = 16 controls; *n* = 21 unruptured aneurysms; *n* = 21 ruptured aneurysms)^70^. This unsupervised analysis correctly ordered samples based on known diagnosis and/or severity (*P* < 0.01; Fig. 5h and Extended Data Fig. 10a). By plotting cell signature scores for key cell types we have found to be aneurysm-associated, we observed an early peak of *POSTN*^*+*^ perivascular fibroblasts that are associated with unruptured aneurysms, whereas *ACP5*^*+*^ *CD68*^*+*^ Mϕs increased later in the disease trajectory and are associated with rupture (Fig. 5i and Extended Data Fig. 10b). Conversely, smooth muscle cells decreased throughout the disease trajectory, consistent with previous reports^60,71^. We confirmed trajectories of smooth muscle cells and *POSTN*^*+*^ perivascular fibroblasts with immunostaining in an independent cohort (*n* = 4 controls; *n* = 5 unruptured; *n* = 5 ruptured; Fig. 5j and Extended Data Fig. 10c, d). Thus, changes in distinct perivascular cell populations that drive fibrosis and inflammation occur throughout the disease progression of brain aneurysms.

## Discussion

Here, we report the cellular landscape and transcriptomic perturbations in human brain aneurysms based on 100,409 single cells/nuclei and 127,254 spatially segmented cells. We uncover cell determinants of pathologic transitions in cerebrovascular structure, define molecular hallmarks of selective cell vulnerabilities, including expression of GWAS risk genes and pathological transcriptional programs in perivascular fibroblasts, and expand the repertoire of recognized infiltrating immune cells in brain aneurysms, such as distinct *ACP5*^+^ macrophages. Taken together, these analyses uncover potential links to disease progression and aneurysm rupture. Data are available for further research at https://brain-aneur.cells.ucsc.edu.

Brain aneurysm formation has been linked to endothelial dysfunction, smooth muscle cell loss and inflammation^6,54,72^. While fibrosis has been observed in brain aneurysms^73^, the cellular mechanisms underlying fibrosis and its contributions to aneurysm pathology have been largely unexplored. We identify activated *POSTN*^*+*^ perivascular fibroblasts that emerge in aneurysm tissues and replace cytoarchitectural locations normally composed of smooth muscle cells^9,10^. *POSTN*^+^ perivascular fibroblasts express genes that drive processes associated with brain aneurysm pathogenesis, such as ECM remodeling, and share molecular features with deleterious fibroblast transitions in other immune-mediated human diseases^54^. By replacing contractile smooth muscle cells, *POSTN*^+^ perivascular fibroblasts may also shift aneurysmal wall mechanics toward collagen-dominated load bearing, increasing stiffness and lowering tensile strength and physiological extensibility, ultimately reducing vascular burst resistance^74–77^; however, future experimental studies are needed to determine whether *POSTN*^*+*^ perivascular fibroblasts contribute to aneurysm development and progression or instead reflect a secondary response to a loss of smooth muscle cells.

Our data suggest *POSTN*^+^ perivascular fibroblasts may derive from quiescent perivascular fibroblasts via induction of *GLIS2*, a zinc finger transcription factor previously implicated in autosomal dominant polycystic kidney disease, which has an elevated risk for brain aneurysms^42–48^. In vivo lineage-tracing studies in mice have shown that perivascular fibroblasts and pericyte-lineage cells can contribute to profibrotic extracellular matrix deposition and undergo myofibroblast-like transitions in response to neurological injury, with relative contributions varying by lesion context^78–86^. Future works using in vivo lineage-tracing approaches are warranted to confirm the cellular origins of *POSTN*^+^ perivascular fibroblasts and the time-course of their appearance in brain aneurysms^87–90^.

Our results also highlight increased communication between vascular and immune cell types beyond the endothelial luminal interface^91^. Fibrosis often results from chronic inflammation^92^, and we identify accumulation of *ACP5*^*+*^ Mϕs in the wall of aneurysms, which are rarely found in the cerebrovasculature or other cerebrovascular diseases, such as arteriovenous malformations^9,10^. Transcriptionally similar macrophages have been reported in other aneurysms, such as those in the aorta^93^. We identify these cells as a major source of secreted vascular destabilizing enzymes and correlate them with brain aneurysm rupture. In other fibrotic human diseases, such as idiopathic pulmonary fibrosis, *ACP5*^*+*^ cells also contribute to the progression of fibrosis^73^. Our findings of a perivascular fibroblast–myeloid cell communication axis support a similar interplay between fibrosis and inflammation in brain aneurysms. Both cell-to-cell communication analyses and cell culture experimental validations nominated MIF–CD74 signaling as a potential mechanism regulating *ACP5*^*+*^ Mϕ accumulation with aneurysm progression, and we postulate that an overlooked perivascular fibroblast-myeloid cell communication axis may be associated with risk for aSAH from brain aneurysms.

Our study is not without its limitations. Fresh cortical arterial segments from the circle of Willis arteries are not obtainable intraoperatively due to risks for stroke. Based on previous benchmarking experiments for gene expression profiling in aneurysm tissues^14,15^, we used a control group composed of small cortical vasculature and postmortem arterial specimens. Thus, it is possible that differences in anatomic location or postmortem conditions may contribute to some alterations in relative cell abundance and/or gene expression. Aneurysm tissue biopsies could only be taken from aneurysms treated with microsurgery and may introduce a selection bias as certain characteristics make aneurysms more suitable for surgical clip ligation. Finally, aneurysm tissue samples are often only several cubic millimeters in size with comparatively limited cell inputs when compared to other sample types undergoing single-cell genomic profiling. Therefore, it remains possible that this study was underpowered to detect pathologically nuanced molecular contributions from rare cell populations and highlights the need for future work in larger, diverse patient cohorts with multi-omic cell-resolution arrays, for example, epigenomics, to delineate further aberrancies that drive disease and/or modify aneurysm morphology.

Nonetheless, our results provide a cell atlas of brain aneurysms, which have no therapeutic option, and treatment is dependent on observation or risky surgical interventions. The molecular blueprint provided by this study substantially extends our mechanistic understanding of brain aneurysms and nominates new cells and pathways with translational promise for the development of therapeutic options to treat this often fatal human cerebrovascular disease.

## Methods

### Ethics statement and tissue acquisition

Adult human brain aneurysm tissue specimens and clinical data were obtained from the University of California San Francisco (UCSF), Barrow Neurological Institute/St. Joseph’s Medical Center, the University of Washington, and the Helsinki University Hospital with protocols approved from the institutional review board and ethics committee. Written informed consent with the purpose of collecting tissues for the purpose of research was obtained before the planned neurosurgical operation or postmortem tissue acquisition. For scRNA-seq experiments, definition and acquisition of control non-aneurysmal cerebrovascular specimens were performed as previously described^9^. In brief, control cerebral cortex was obtained as part of a neurosurgical operation to reach deep seated lesions (>2 cm from the cortical surface) causing epilepsy. Isolated cortical samples had no radiographic abnormalities on magnetic resonance imaging, showed no abnormalities on intraoperative electrocorticography and displayed no apparent abnormalities on rapid hematoxylin and eosin staining. In total, five donor non-aneurysmal control specimens were obtained and included in scRNA-seq experiments. Additional snap-frozen posterior cerebral artery samples obtained from the proximal P2 segment were obtained from snap-frozen, postmortem coronal brain sections and included for snRNA-seq experiments (*n* = 3 donors) or spatial transcriptomics (*n* = 1 donor). Finally, for control specimens for immunostaining experiments snap-frozen, postmortem middle cerebral artery specimens were obtained from the M1 segment (*n* = 4 donors).

For aneurysm tissue specimens (total *n* = 18 donors), diagnosis of human brain aneurysms were angiographically confirmed preoperatively. For all experiments, fresh tissues obtained as part of planned microsurgical clip ligation or bypass. All included aneurysms were without concomitant vascular malformation. Tissues were obtained with close collaboration with neurosurgeons to minimize tissue disruption, including the avoidance of electrocautery. Fresh tissues were immediately processed or snap frozen in liquid nitrogen for scRNA-seq (*n* = 2 donors) or other analyses (snRNA-seq (*n* = 6 donors), spatial transcriptomics (*n* = 1 donor) and immunostaining (*n* = 10 donors)), respectively. Demographic information for all human tissues utilized in the present study are summarized and broken down by experiment in Supplementary Table 1.

### Isolation of brain aneurysm tissue specimens

Following microsurgical clip ligation or bypass to exclude the aneurysm from the cerebral circulation, residual aneurysm tissues were then sharply dissected and excised with surgical microscissors under high magnification with a Zeiss KINEVO 900 operative microscope (Carl Zeiss). This yielded several cubic millimeters of tissue. Aneurysm tissue specimens were placed in chilled, oxygenated Dulbecco’s Modified Eagle Medium (DMEM; Thermo Fisher Scientific) and immediately transported to the laboratory on ice. All tissue processing and cell isolations were performed in a class II biological safety cabinet with autoclave-sterilized equipment. Tissues were washed in chilled, oxygenated DMEM three times to remove contaminating or circulating cells. Aneurysm tissue was cut into ~1-mm pieces and scored with a scalpel. Aneurysm tissues were placed in chilled, oxygenated DMEM on ice and immediately processed to create single-cell suspensions.

### Creation of control and brain aneurysm cell suspensions

Dissected aneurysm tissues and non-aneurysmal control cerebrovascular specimens were dissociated using a technique previously designed and benchmarked to isolate vascular cells from large arterial pathologies, such as arteriovenous malformations, as we have previously reported^9^. Briefly, tissues were incubated for 45 min in 0.2% Collagenase Type 2 (Worthington Biochemical Corporation) diluted in oxygenated DMEM at 37 °C with gentle agitation in an Eppendorf LoBind 5-ml tube (Eppendorf North America). Cell suspensions were filtered through a sterile 40-μm cell strainer (Fisher Scientific) to isolate undigested debris. Cells were collected with centrifugation at 500*g* for 5 min, the supernatant aspirated, and then incubated in Gibco ACK lysing buffer (Thermo Fisher Scientific) for 3 min at room temperature to remove residual erythrocytes. Cells were then washed and resuspended three times in sterile RNase-free phosphate-buffered saline (PBS) (Invitrogen) containing 0.04% bovine serum albumin (Sigma-Aldrich). A 10 μl aliquot of the cell suspension was mixed 1:1 with 0.4% trypan blue (Thermo Fisher Scientific) and cells were counted on a hemocytometer to confirm viability and yield. For scRNA-seq experiments, we used five non-aneurysmal control tissue samples and two aneurysm tissue samples (Supplementary Table 1).

### Creation of control and brain aneurysm nuclear suspensions

Snap frozen aneurysm tissues (*n* = 6 donors) and non-aneurysmal, postmortem posterior cerebral artery specimens (*n* = 3 donors) were dissociated employing an adapted protocol modified from previous publications^10^, specifically tailored for the isolation of cells from small, low cell input arterial samples. All dissociation steps were performed as rapidly as possible on ice. Aneurysm and arterial tissue (≥10 mg) was thawed on ice for 5 min with 1 ml of extraction buffer (ExB): Nuclei EZ Prep Lysis Buffer (Sigma, NUC101), 0.2 U ml^−1^ RNase inhibitor (Sigma 3335399001), EDTA-free protease inhibitor cocktail (Roche, 11873580001) and 5% kollidon VA6 (BASF Pharm 25086-89-9). Tissues were minced with microscissors and homogenized with a 2-ml glass Dounce grinder (Sigma, D8938) until no visible tissues were left. Homogenates were then passed through a pre-wetted 40-mm cell strainer and flow through collected in a 50-ml LoBind conical tube (Eppendorf, 0030122232). Cell strainers were then washed and flow through collected with wash buffer (WB): 2% BSA containing 0.04 U ml^−1^ RNase inhibitor and EDTA-free protease inhibitor. Liberated nuclei were pelleted at 500*g* for 6 min with brake on 1′, resuspended in 3 ml of wash buffer, passed through cells strainers caps atop FACS tubes and pelleted again. Clean nuclei were then stained with 7-AAD (1:100, Sigma SML1633) in 200 μl of samples buffer (SB): 2% BSA containing 0.2 U ml^−1^ RNase inhibitor, EDTA-free protease inhibitor. Stained nuclei were pelleted at 500*g* for 6 min, resuspended in 100 ml of SB, sorted in a BD FACS Aria II Flow Cytometer (BD Biosciences) and collected in 1 ml of SB without protease inhibitor. In pilot experiments, we noticed that the cytometer overestimated nuclei count by a factor of 4, and we therefore targeted around 40,000 nuclei to target ~10,000 nuclei per sample whenever possible.

### Single-cell RNA sequencing

Fresh, whole cell suspensions were loaded at approximately 10,000 cells per reaction based on hemocytometer cell counts into a single lane of a 10x Genomics Chip and inserted into the Chromium Controller (10x Genomics). Single-cell sequencing libraries were performed with Chromium Single Cell 3′ Reagent kits v2 and v3 as described by the manufacturer (10x Genomics). The quality of the libraries generated was confirmed with an Agilent 4200 TapeStation System (Agilent Technologies). Libraries were sequenced with an Illumina NovaSeq 6000 (Illumin) with a targeted sequencing depth of 50,000 reads per cell.

### Single-nucleus RNA sequencing

Nuclei across conditions were pooled into two batches with a target of 10,000 nuclei per reaction. Single-nuclei sequencing libraries were performed with Chromium Single Cell 3′ Reagent kits as described by the manufacturer (10x Genomics). Quality of the libraries generated were confirmed with an Agilent 4200 TapeStation System (Agilent Technologies). Libraries were sequenced with an Illumina NovaSeq 6000 (Illumina) with a targeted sequencing depth of 50,000 reads per nucleus.

### Slide-seq spatial transcriptomics

Snap frozen aneurysm (*n* = 1 donor) or control posterior cerebral artery tissues (*n* = 1 donor) were embedded in Tissue-Tek optimal cutting temperature compound (Sakura Finetek USA), cryosectioned at a thickness of 10 μm, and mounted on 3 × 3-mm spatially indexed bead Seeker Tiles (Curio Biosciences). Slide-seq spatial transcriptomic libraries were then prepared using the Curio Seeker Kit as described by the manufacturer (Curio Bioscience). Quality of the libraries generated were confirmed with an Agilent 4200 TapeStation System (Agilent Technologies). Libraries were sequenced with an Illumina NovaSeq 6000 (Illumina) with a targeted sequencing depth of 100,000 reads per bead.

### 10x Genomics Xenium spatial transcriptomics

Postmortem FFPE brain aneurysm specimens were sectioned at a thickness of 5 μm and floated on a warm water bath. Sections were then scored to remove as much of the excess paraffin wax surrounding the tissue before being positioned onto the Xenium slide. Care was taken to avoid covering any of the fiduciary markings on the Xenium slide by either the tissue section or the remaining paraffin wax. Completed slides were then placed into slide mailers, sealed with parafilm, and stored in a desiccator at room temperature for no more than 4 weeks before processing on the 10x Genomics’ Xenium Analyzer. Xenium slides were prepared strictly according to the manufacturer’s specifications, including the add-on protocol for the Xenium Multi-Tissue stain. Briefly, Xenium slides were incubated with the resuspended custom gene expression probe buffer and allowed to incubate overnight. Following the washout of unbound probes, ligation was performed to circularize the hybridized probes, and rolling circle amplification was carried out to amplify the gene-specific barcodes. Next, cell boundaries and interiors were stained with the add-on cell segmentation antibodies overnight. Finally, the autofluorescence was quenched and DAPI was incubated overnight before final processing on the Xenium Analyzer. The onboard Xenium Analyzer instrument software (v.3.3.0.0) performed transcript decoding, cell segmentation calls, and quality control calculations, which are then exported for further downstream bioinformatic analysis.

### Immunofluorescent staining

Snap-frozen tissue sections were embedded in Tissue-Tek optimal cutting temperature compound (Sakura Finetek USA), cryosectioned at a thickness of 14 μm, and collected on SuperFrost Plus Microscope Slides (Fisher Scientific). Tissue sections were stored at −80 °C until used for immunostaining. Immunostaining experiments were performed on non-aneurysmal postmortem middle cerebral artery tissues as control (*n* = 4 donors) and aneurysm tissue samples (*n* = 10 donors) unless otherwise specified and stratified based on rupture status. Supplementary Table 1 shows donor demographic information. Tissue sections were allowed to equilibrate at room temperature for 30 min and then immersion fixed with 4% paraformaldehyde (Sigma-Aldrich) for 15 min at room temperature. Tissue sections were then blocked with PBS containing 10% donkey serum (Sigma-Aldrich) and 0.2% Triton (Sigma-Aldrich) for 1 h at room temperature and incubated in primary antibody overnight at 4 °C. Primary antibodies included goat anti-human podocalyxin (1:100 dilution, R&D Systems), mouse anti-human calponin-1 (CNN1; 1:100 dilution, MilliporeSigma), rabbit anti-human COL1A1 (E8F4L) (1:100 dilution, Cell Signaling Technology), mouse anti-human COL1A1 (E3E1X) (1:100 dilution, Cell Signaling Technology), rabbit anti-human periostin (E5F2S) (1:100 dilution, Cell Signaling Technology), mouse anti-human TRAcP (clone 9C5, 1:100 dilution, MilliporeSigma), COL3A1 (ab7778) (1:250 dilution, Abcam) and rabbit anti-human CD68 (D4B9C) (1:100 dilution, Cell Signaling Technology). Sections were washed with PBS containing 0.05% Triton and incubated with AlexaFluor-conjugated secondary antibodies for two hours at room temperature, unless otherwise indicated. Donkey anti-goat AlexaFluor 488 (1:100 dilution, Thermo Fisher Scientific), donkey anti-rabbit AlexaFluor 555 (1:100 dilution, Thermo Fisher Scientific) and donkey anti-mouse AlexaFluor 647 (1:100 dilution, Thermo Fisher Scientific) were used to detect goat, rabbit, and mouse primary antibodies, respectively. For experiments utilizing mouse anti-human COL1A1 (E3E1X), COL1A1 was detected with incubation in biotin-conjugated donkey anti-mouse antibody (1:100 dilution, Thermo Fisher Scientific) for 2 h at room temperature followed by a 1-h incubation with DyLight 649 conjugated streptavidin (1:100 dilution, Vector Laboratories) at 37 °C. All sections were washed and then incubated with 1% Sudan Black for 10 min at room temperature to quench endogenous autofluorescence. Slides were mounted using DAPI-containing Vibrance anti-fade mounting medium (Vector Laboratories) and allowed to cure overnight at room temperature. Primary antibodies were confirmed for target specificity by the manufacturer before use.

### Histological staining

Formalin-fixed paraffin-embedded tissue sections were sectioned at a thickness of 5 μm, deparaffinized with xylene and rehydrated with distilled water. Masson Trichrome staining was performed using the Trichrome Stain kit (Abcam). Slides were placed in preheated Bouin’s Fluid for 60 min, followed by a 10-min cooling period. Tap water was used to rinse the slides until the section was completely clear, followed by a rinse in distilled water. To stain nuclei, slides were incubated in Weigert’s iron hematoxylin for 5 min, followed by a rinse in running tap water for 2 min. Biebrich Scarlet/Acid Fuchsin solution was applied to the sections for 15 min, followed by a rinse in distilled water. Slides were then incubated in phosphomolybdic/phosphotungstic acid solution for 15 min. Without rinsing, Aniline Blue solution was applied to the sections for 5–10 min, followed by a rinse in distilled water. Slides were then incubated in 1% acetic acid solution for 3–5 min and immediately dehydrated in two changes of 95% ethanol, followed by two changes of 100% ethanol. Clearing of sections was performed using xylene and mounted using synthetic resin. Light microscopy imaging was performed using the Keyence BZ-X1000 (Keyence) with a ×20 objective. Percentage of fibrosis was determined using a method previously described^94^.

### Confocal image analysis

All confocal image analysis was performed with a Nikon Eclipse Ti2 confocal microscope (Nikon) with an air-immersion ×20 objective. For quantitative imaging experiments, a minimum of five randomly selected image fields were obtained in three nonadjacent tissue sections per individual at least 100 µm apart. For each field, a 1–12-mm *z*-stack image was obtained. Maximum projection *z*-stack images were subsequently reconstructed with National Institutes of Health (NIH) ImageJ software. No tissues were excluded from analysis and a minimum group size of five donors per condition were used for each analysis. To quantify the number of PODXL-, CNN1-, COL1A1-, POSTN- or TRAcP-positive cells, positively stained cell bodies were counted as defined by DAPI-positive nuclei with the NIH ImageJ multipoint tool and expressed as number of positive cells per mm^2^ of tissue. Statistical analyses with a Student’s two-sided *t*-test or one-way ANOVA with Tukey’s multiple comparisons test were performed for two groups or >2 groups using GraphPad Prism (Dotmatics), respectively. All data are plotted as mean ± s.e.m. with individual data points shown.

### Proximity ligation assay

PLA was used to determine protein–protein interactions in FFPE tissue using Duolink PLA kit (Sigma-Aldrich), following the protocol as previously described^95,96^, with some modifications. Primary antibodies used were MIF mouse antibody clone 2A10-4D3 (5 µg ml^−1^, WH0004282M1, Sigma-Aldrich), CD74 rabbit antibody (5 µg ml^−1^, HPA010592, Sigma-Aldrich) and the CD68 goat antibody (5 µg ml^−1^, PA5-143237, Invitrogen). Primary antibodies were confirmed for target specificity by the manufacturer before use. FFPE sections of 5-µm thickness were first baked in an oven at 65 °C for 30 min and then were deparaffinized using 2 × 5-min washes with xylene, 2 × 5-min washes with 100% ethanol, subsequently the sections were rehydrated with serial washes of 5 min with 95%, 90%, 80% and 70% ethanol and then a 5-min wash with 1× PBS before moving to antigen retrieval. All sections were treated with an antigen retrieval step to enhance the antibody signal in the fixed tissue. In brief, 1× Target Retrieval Solution (S236784, Agilent-Dako) was preheated to 65 °C and the sections were placed in it overnight at 65 °C. The slides were then cooled to room temperature for 15 min and washed twice in 1× PBS and then proceeded with the assay. Sections were blocked with the provided blocking solution at 37 °C for 60 min. Primary antibodies were diluted in the provided antibody diluent and incubated at 4 °C overnight. The sections were then washed with buffer A at room temperature (3 × 5 min). Proximity probes were diluted in the same antibody diluent that was used for the primary antibodies and was applied to the sections for 60 min at 37 °C. Unbound probes were removed by washing the sections with buffer A (3 × 5 min) at room temperature. Sections were incubated with ligation solution for 30 min at 37 °C, then washed with buffer A (3 × 5 min) at room temperature. The rolling amplification-hybridization mixture was then added for 100 min at 37 °C. The sections were washed with buffer B (2 × 10 min) and buffer A (1 × 1 min) at room temperature. After this we proceeded with counterstaining with secondary antibody anti-goat IgG H&L AlexaFluor Plus488 (1:500 dilution, Invitrogen) and incubate for 30 min at 37 °C. The sections were then washed with buffer A (1 × 2 min) and (0.01×) buffer B (1 × 1 min) at room temperature. Add 1% Sudan Black B diluted in 70% ethanol and incubate for 20 min at room temperature to quench autofluorescence. The sections were then washed with buffer B (2 × 10 min) and (0.01×) buffer B (1 × 1 min). The sections were dried and mounted using provided mounting medium containing DAPI. These sections were then visualized using Nikon eclipse Ti2 confocal microscope.

### Cell culture

#### Culture of primary human brain vascular adventitial fibroblast and human brain vascular smooth muscle cells

Primary human brain vascular adventitial fibroblasts (‘fibroblasts’) and primary human brain vascular smooth muscle cells (‘smooth muscle cells’) were obtained from ScienCell. Both these cells were cultured in poly-L-lysine-coated T-75 flasks using Fibroblast Medium (2301, ScienCell) and Smooth Muscle Cell Medium (1101, ScienCell) containing supplements each. Primary human macrophages were obtained from Celprogen (36070-01) were cultured in T-75 flasks coated with extracellular matrix from Celprogen (36070-01-T-75) in medium containing serum from Celprogen (M36070-01S). All cells were maintained at 37 °C in 5% CO_2_.

#### Transfection of primary human brain vascular adventitial fibroblast and human brain vascular smooth muscle cells

When the fibroblasts or smooth muscle cells were 75% confluent the cells were seeded in a poly-L-lysine-coated six-well plate (5 × 10^5^ cells per well) and incubated in medium for 18 h. A mammalian expression vector encoding GLIS2–GFP-tagged overexpression was driven by the EF1a promoter and was synthesized by VectorBuilder (vector ID VB250505-1325dbu). An empty backbone vector–GFP-tagged served as a negative control. The cells were transfected with 2 µg of plasmid DNA using FuGENE 4K transfection reagent (4K-1000) according to the manufacturer’s instruction. Transfected cells were collected by incubating the cells in Accutase (Innovative Cell Technologies) for 5 min at 37 °C, 48 h post-transfection for robust expression of transfected protein. To determine transfection efficiency, we compared cells with GFP–control vector and GFP–Glis2 overexpression vector. The cells were washed with PBS (1×), then fixed using 4% ice-cold paraformaldehyde (Thermo Scientific) for 30 min at room temperature followed by three washes with PBS (1×). The nuclei were stained using DAPI (1:1,000 dilution, Invitrogen).

#### Macrophage treatment with macrophage migration inhibitory factor

Primary human macrophages were cultured as described earlier. Once the cells reached 75–80% confluency they were seeded (2 × 10^5^ cells per well) on a six-well plate coated with extracellular matrix (E36070-01-6 well) and weaned off serum medium and replaced with serum-free medium from Celprogen (M36070-01). We first treated cells with interferon (IFN)-γ (PeproTech) at a concentration of 100 ng ml^−1^ for 24 h to ensure CD74 expression as previously described^97,98^. Next, we added anti-CD74 antibody (MedChemExpress) at concentration of 5 µg ml^−1^ and incubated for 30 min followed by washing with medium. Finally, we added human MIF recombinant protein (PeproTech) at a concentration of 50 ng ml^−1^ and incubated for 8 h.

#### Quantitative PCR with reverse transcription

Cells were collected using Accutase. Cellular RNA was extracted using TRIzol–chloroform and converted to first-strand cDNA using qScript cDNA Synthesis kit (95047-100, QuantaBio). Quantitative PCR with reverse transcription (RT–qPCR) was performed using a LightCycler 480 (Roche) with FisherBrand SYBR green qPCR kit (A59529, FisherBrand) and primers were added at a 500 nM final concentration. The primer sequences used are listed in Supplementary Table 6. Human *Actin* was used as an internal reference gene. FC values were calculated using the 2^−ΔΔCt^ method.

### Cell-resolution bioinformatic data analysis

#### scRNA-seq data processing

scRNA-seq FASTQs were aligned and quantified with STARSolo v.2.7.10a with specific parameters for multi mapping with expectation-maximization, intron-inclusion, cell barcode and unique molecular identifier (UMI) correction: --soloCBmatchWLtype 1MM_multi_Nbase_pseudocounts --soloUMIdedup 1MM_CR --soloFeatures GeneFull_Ex50pAS Velocyto --soloMultiMappers EM --clipAdapterType CellRanger4 --soloUMIfiltering MultiGeneUMI_CR^99^. Reference genome annotation used is GENCODE 39 with modifications listed by 10x Genomics at https://support.10xgenomics.com/single-cell-gene-expression/software/release-notes/build#GRCh38_2020A. Low-quality cells were excluded if any of the following criteria were met: <1,000 genes per cell, predicted doublet with scds’ hybrid mode^100^, or a maximum mitochondrial content >10 %.

#### snRNA-seq data processing

snRNA-seq FASTQs were aligned and quantified as described above with STARSolo (v.2.7.10a)^99^. Genotype demultiplexing was then performed using the Vireo pipeline and cellsnp-lite for calling single-nucleotide polymorphisms^101^. Genotype free demultiplexing was carried out utilizing single-nucleotide polymorphisms from the 1000 Genomes Project whitelists filtered on .0001 MAF. Aligning the donors with Vireo was conducted by calculating the least genotype difference using genotype probability. Specific donors were demultiplexed based on variant correlation based on intersected variants between any two donors between different single-nucleus reaction pools utilizing deconvolved donors from Vireo. The workflow for Vireo deconvolution was conducted as laid out in their example workflow for genotype free donor matching at https://github.com/single-cell-genetics/vireo/blob/master/examples/donor_match.ipynb. Following demultiplexing, low-quality nuclei were excluded if any of the following criteria were met as described above: <1,000 genes per nucleus, predicted doublet with scds’ hybrid mode^100^ or a maximum mitochondrial content >10%. Nuclei not called to donors following demultiplexing were also excluded from subsequent analyses.

#### Integration, clustering and annotation

Seurat’s SCTransform v2 workflow was used for normalization set to 3,000 most variable genes followed by filtering for mitochondrial, ribosomal genes and MALAT1^102^. The residuals were input into principal-component analysis (PCA) and batch corrected with Harmony over 30 principal components with theta = 0.575 over the 10x batch metadata to create an integrated Seurat object^16^. UMAP dimensional reduction was conducted on the Harmony-corrected components with min.dist = 0.3. De novo clustering was conducted with the original Louvain algorithm with the resolution set to 2 to capture rare immune cell subtypes. Marker genes for the unannotated clusters were computed using a Wilcoxon rank-sum test implemented with Seurat’s built-in ‘FindAllMarkers’ function. A ‘weighted specificity score’ was calculated as avg_log_2_FC × |pct.1 − pct.2| and was used to sort marker genes in descending order of importance. Manual curations of cluster annotations were performed using established control cerebrovascular atlases^9–12^. To refine fibroblast cluster annotations due to the observed transcriptional continuum, we used UCell scores (v.2.2.0) using the top 20 marker genes ranked by the weighted specificity score to guide annotations^103^. Annotated cluster markers and weighted specificity scores were calculated using the same function and parameters as described previously for the unannotated clusters.

#### Cell proportion analysis

Propeller (v.0.0.3) is a linear modeling-based approach for testing cell proportion differences that leverages biological replicates to estimate biological variability in cell proportions^104^. Here, Propeller was utilized to compare cell state proportions across aneurysm and control samples. To remove the confounding effects of variable parenchymal contamination across samples, cell contributions from neurons, astrocytes, oligodendrocytes and oligodendrocyte precursor cells were censored for the purposes of the proportions analysis. Transformed proportions were calculated across all samples for the remaining vascular and immune cell types using the arcsin-square root method. A *t*-test with Benjamini–Hochberg correction was performed between the aneurysm and control samples using the propeller.ttest function on default settings.

#### Differential gene expression analysis

Differential gene expression analysis was performed using the DESeq2 ‘wald’ algorithm for endothelial cells, smooth muscle cells, fibromyocytes and perivascular fibroblasts pseudobulked at the donor level^105^, in accordance with recently published best practices on combating false discovery in differential expression analysis^106^. We incorporated preparation (cell versus nuclei) as a covariate in our model and employed logarithmic FC shrinkage using the ‘apeglm’ R package (v.1.26.1)^107^. Control endothelial cells were sorted in silico to select cells expressing an enriched arterial UCell signature before differential gene expression analysis and compared to aneurysm endothelial cells. Arterial UCell signatures were calculated using the top 30 arterial marker genes identified in our previous single-cell atlas of the control cerebrovasculature^9^. Statistical significance was assessed using the following threshold to call differentially expressed genes (DEGs): Benjamini–Hochberg-corrected *P* < 0.05 and |avg_log_2_FC| > 1.

#### Transcriptome-wide analysis of differential expression

Univariate TRADE was implemented using the results of the pseudobulked per-cell-type DESeq2 model described above, as outlined in the tutorial released by the authors^24^. Jack-knifing was carried out by iteratively dropping one sample at a time and repeating the TRADE analysis to calculate the standard errors of TRADE estimates per cell type. For cell types that are not well-conserved between aneurysms and controls (for example, activated macrophages), they were excluded from the TRADE analysis.

#### Gene Ontology analysis

Gene Ontology analyses were performed using Single Cell Pathway Analysis (SCPA) (v.1.6.1) to investigate enriched biological processes unless otherwise indicated^108^. In brief, SCPA identified pathway associations by assessing changes in a multivariate distribution of any given pathway across conditions instead of assessing the over-representation of the underlying genes. For SCPA, SCT-transformed matrices were extracted as input. GO_Biological Processes were used as the pathways for comparison. SCPA was run on default parameters and significant pathways was assessed using the following thresholds: Bonferroni-corrected *P* < 0.01 and |FC| > 1.

#### GWAS integration

Integration of the aneurysm GWAS dataset with the single-cell dataset was first performed using scPagwas (v.1.3.0)^33^. In brief, scPagwas performed a polygenic regression of GWAS signals on activated pathways found in a scRNA-seq dataset to prioritize trait-relevant cell populations. GWAS summary statistics were obtained from Bakker et al.^28^. Built-in hg37 block annotations, KEGG pathways and chromosome LD references were utilized from the scPagwas package. SCT-transformed values were inputted and bootstrap iterations were set to 10. Benjamini–Hochberg *P* value correction was performed within the scPagwas pipeline.

For the MAGMA analysis, we leveraged MAGMA:CellTyping (v.2.0.8)^32,109^ to integrate GWAS summary statistics data with our aneurysm cell atlas. GWAS summary statistic from Bakker et al.^28^ was formatted using MungeSumStats (v.1.15.1)^110^, performed using default MAGMA settings. For each trait, common risk variants that reached genome-wide significance were identified and mapped to their respective genes using the default MAGMA parameters. Adjusted *F* statistics were then computed using a multiple linear principal components regression model to derive significance values for each gene. Specificity scores were then generated for each gene from the cell annotations described in our cell type dataset (CTD) to achieve cell type enrichment. The CTD was derived using the expression matrix of our single-cell Seurat object (genes as rows and cells as columns) and cell annotations listed in the metadata. *P* values per cell type were then Benjamini–Hochberg-corrected and only significant cell type enrichments were highlighted.

#### Fibroblast activation and pseudotime analysis

Perivascular fibroblast populations were sorted in silico and subsetted. Marker genes for an activated fibroblast population were obtained from Chaffin et al.^35^. Top 20 markers ranked in descending order of limma-voom logFC were selected for calculating a UCell-activated fibroblast signature. Pseudotime analysis was performed using Monocle3 (v.1.3.4) on the subsetted Seurat object. The Seurat object was converted to cell_data_set format using the SeuratWrappers package (v.0.3.3) with the same UMAP embeddings retained. To identify highly specific gene modules that changed across pseudotime, a clustering resolution of 4 × 10^−2^ was selected to produce 35 gene modules. From this, two modules representing the early and late phenotypes each were identified based on their specificity to the pseudotime boundaries defined between fibroblasts, activated fibroblasts and myofibroblasts. Module genes were inputted into enrichR (v.3.2) using the ‘GO_Biological_Process_2023’ database to determine enriched biological processes^111^. For visualization of the module expression over pseudotime, module gene names were passed into UCell to calculate a module signature score.

#### CellChat communication analysis

CellChat (v.2.1.2) is an approach for inferring cell–cell interactions based on the expression of ligand–receptor pairs in scRNA-seq datasets^49^. Here, CellChat was performed in the comparison analysis mode between control and aneurysm cells. SCT-transformed matrices were inputted for both conditions. The CellChatDB search was restricted to ‘secreted signaling’ communications. Communication probabilities were calculated using the ‘triMean’ method with population size and gene expression projection onto PPI turned off. Communications were filtered to those found in a minimum of ten cells. All other steps were carried out using default parameters. Most visualizations were created using the default toolkits provided by the authors. To visualize the differential ingoing and outgoing interaction strengths between aneurysm and condition, values for each cell type’s ingoing and outgoing interaction strengths were computed individually between conditions and subtracted from each other.

#### Scissor analysis

Scissor deconvolution analysis (v.2.0.0) was carried out to map phenotypic changes found in the bulk RNA-seq dataset of aneurysms and control cerebrovasculature onto our scRNA-seq dataset^68^. Scissor was run according to the tutorial using the rupture status (unruptured versus ruptured) as the phenotype of interest. The α parameter was identified using a grid search with a cutoff parameter of 0.08 and cells were identified by Scissor using the binomial (logistic regression) test. A reliability test was performed to confirm the high fidelity of Scissor-selected cells (*P* < 0.01). To assess the statistical significance for the enrichment of Scissor-selected cells, a hypergeometric test was performed and FC was calculated as the ratio between observed and expected enrichments. Differential gene expression analysis was calculated between rupture^+^ aMϕs and nonselected aMϕs using the same differential gene expression analysis method utilizing the DESeq2 algorithm as described above. Statistically significant DEGs were identified using the same stringent criteria as previously described: Benjamini–Hochberg-corrected *P* value < 0.05 and |avg_log_2_FC| > 1. Gene Ontology for the top statistically significant DEGs was then performed using enrichR (v.3.2) using the ‘GO_Biological_Process_2023’ database^111^.

### Bulk RNA-seq bioinformatic data analysis

#### Bulk RNA-seq data acquisition and processing

Raw sequencing data were obtained from Kleinloog et al.^7^ from the Gene Expression Omnibus (accession code GSE122897)^7^. Of note, this dataset contained bulk RNA-seq from 43 intracranial aneurysm and 16 control cortical artery samples (*n* = 59 total). Rupture status of one aneurysm was undetermined and was excluded from analyses that required knowledge of rupture status. Bulk RNA-seq data were aligned on the same indexed transcriptome of StarSolo as described above^99^.

#### Bulk RNA-seq cell deconvolution

BayesPrism (v.2.2.2) is a Bayesian model that can infer both cell type fractions and gene expressions from bulk RNA-seq datasets by using a scRNA-seq dataset as per ref. ^22^. Here, the Kleinloog dataset was deconvoluted using our scRNA-seq dataset as the ref. ^7^. SCT counts were inputted from the single-cell dataset. Due to transcriptional similarities, pvMϕs, monocytes, aMϕs, conventional dendritic cells (cDCs) and microglia (MG) were grouped together as ‘myeloid’ at the level of cell type but registered separately as cell states. All cell types from the single-cell dataset were retained for the deconvolution step. BayesPrism was executed on default parameters (outlier.cut = 0.1, outlier.cutoff = 0.01) and the reference count matrix was filtered for ribosomal, mitochondrial, MALAT1 and sex genes before input. Deconvoluted cell proportions were obtained using the ‘first’ theta parameter and cell states layer. Owing to variable parenchymal contamination, cell contributions from neurons, astrocytes, oligodendrocytes and oligodendrocyte precursor cells were excluded and the remaining cell state proportions were re-calculated across the sum of proportions left remaining for each sample. PropRatio was calculated using the mean proportion for each cell state by condition. A two-sided Student’s *t*-test was used to assess an FDR-corrected *P* value for deconvoluted cell state differences between conditions. For benchmarking of BayesPrism parameters, pseudobulks were aggregated at the level of samples. Samples with bulk RNA-seq were pseudo-aligned to a reference human transcriptome (GRCh38 assembly, Ensembl v.96) using Kallisto (v.0.46.2) with the bootstrapping number set to 100 and aggregated into count matrices using sleuth (v.0.30.1)^112,113^.

#### Disease severity pseudotime

Disease severity pseudotime analysis was performed based on a modified protocol from Mukherjee et al.^70^ where we performed UMAP embeddings of individual bulk RNA-seq samples and then performed lineage inference using Monocle3 (v.1.3.4)^39^. The pre-filtered bulk RNA-seq matrix from Kleinloog et al. was assembled into a Seurat object, SCT-transformed and embedded using Seurat’s runPCA and runUMAP functions using the parameter of min.dist = 0.01. Pseudotime analysis was then performed using Monocle3 (v.1.3.4). To calculate cell type changes across the disease pseudotime, the top 25 annotated cluster markers ranked in descending order by the weighted specificity score were used to compute a cell type-specific UCell signature score for each sample and plotted across the pseudotime axis.

### Curio slide-seq spatial transcriptomics bioinformatic analysis

Curio Seeker pipeline was used to align raw FASTQs where reads were trimmed to 50 bp for the barcoded R1 and then 55 bp for the R2 cDNA reads. Prebuilt GRCh38 STAR genome references provided by Curio Biosciences were used to align reads and to generate Seurat objects. Barcoded beads without any reads were filtered. Processed spatial transcriptomics objects were visualized using Seurat’s SpatialFeaturePlot function. A max. cutoff of 99th percentile was applied in instances where outliers dominate the color scaling. Marker genes for an activated fibroblast population were obtained from Chaffin et al.^35^. Top 20 markers ranked in descending order of limma-voom logFC were selected for calculating a UCell-activated fibroblast signature as described above.

### 10x Genomics Xenium in situ spatial transcriptomics bioinformatic analysis

#### Xenium gene panel curation

We designed an aneurysm-tailored 100-gene panel that leverages both single-cell-driven approaches, such as Spapros^114^, and manual literature curation. Attention was given to ensuring redundant coverage of the cell type gene markers first described in our single-cell reference, as well as DEGs, fibrosis activation genes and aneurysm risk genes. Candidate genes were then assessed for compatibility using the 10x Genomics Xenium Panel Designer software (accessed 24 February 2025, for the Xenium v1 chemistry kit) to safeguard against optical crowding before finalizing the 100-gene panel.

#### Cellular segmentation and quality control

The onboard cell segmentation performed by the 10x Genomics’ Xenium In Situ software (v.3.3.0.0) was imported into Seurat (v.5.1.0) for spatial transcriptomics analysis in R (v.4.4.1). Empirically, a threshold of nine transcripts was selected to discard low-quality cell segmentations. SCTransform normalization (v.0.4.1) was deployed with the number of RNA transcripts set as the variable to regress due to large fluctuations in transcript detection across segmented cells. Standard single-cell processing techniques, such as PCA, UMAP embedding, neighbor detection, original Louvain clustering and Propeller proportion analysis, were used as previously described in our single-cell section above. Cluster annotations were based on the top cell type marker expression, and the same analytic frameworks first employed for the single-cell analyses were repeated for the Xenium dataset.

#### Nearest neighborhood spatial analysis

A two-dimensional ranged nearest neighbors search (*r* = 500 μm) was performed using the ‘nn2’ function in RANN (v.2.6.1) package. We used *k* = 15,000 as an upper bound on the number of neighbors to ensure that all possible nearest neighbors within the search radius were captured. With the origin set as the set of all activated perivascular macrophages, neighboring cells were then assigned into 1-µm bins, ranging from 1 µm to 500 µm, and their frequencies were tabulated and sorted by cell type identity. Self-neighbors (bin at 0 µm) were excluded for clarity in the analysis and the mean of the distribution was used to calculate cell-type distances relative to activated perivascular macrophages.

### Statistics and reproducibility

Sample sizes for the single-cell analyses were informed by power calculations performed using scPower^115^. All successfully sequenced biological specimens were included in the analysis and are detailed in Supplementary Table 1. All image quantifications were performed with an investigator blinded to the underlying tissue type (control versus aneurysm). Paired with multiple tiers of validation, the principal findings should be reproducible across multiple aneurysm samples sequenced and stained in the same manner. Pearson’s correlations were calculated for evaluating the replicability of measured bulk RNA-seq cell proportions and simulated pseudobulk cell proportions from the same aneurysm specimens. Representative images in Extended Data Figs. 7f,g and 10c,d were from one of at least three independent experiments with similar results. All statistical tests were two-sided, unless otherwise noted.

### Reporting summary

Further information on research design is available in the Nature Portfolio Reporting Summary linked to this article.

## Online content

Any methods, additional references, Nature Portfolio reporting summaries, source data, extended data, supplementary information, acknowledgements, peer review information; details of author contributions and competing interests; and statements of data and code availability are available at 10.1038/s41593-026-02326-9.

## Supplementary information

Supplementary Information Supplementary Figs. 1–3.

Reporting Summary

Supplementary Tables Supplementary Tables 1–6.

## Data Availability

Data are available to explore via the interactive web-based cell viewer at https://brain-aneur.cells.ucsc.edu/. Sequencing data have been deposited at dbGAP under the accession code phs002624.v6.p1.

## Code availability

Custom code utilized for bespoke analyses is available at https://github.com/jwangbio/aneurysm-analysis.
