# Supplementary material for: Cerebrovascular vulnerability and fibrosis in human brain aneurysms
Source: Nat Neurosci. 2026 Jun 10;29(8):1814–25. doi: 10.1038/s41593-026-02326-9 (PMC13433319; doi:10.1038/s41593-026-02326-9)
Supplement: Supplementary file 1 [file 41593_2026_2326_MOESM1_ESM.pdf]

nature neuroscience

Supplementary information

https://doi.org/10.1038/s41593-026-02326-9

Cerebrovascular vulnerability and fibrosis in human brain aneurysms

In the format provided by the authors and unedited

Supplementary Figure 1

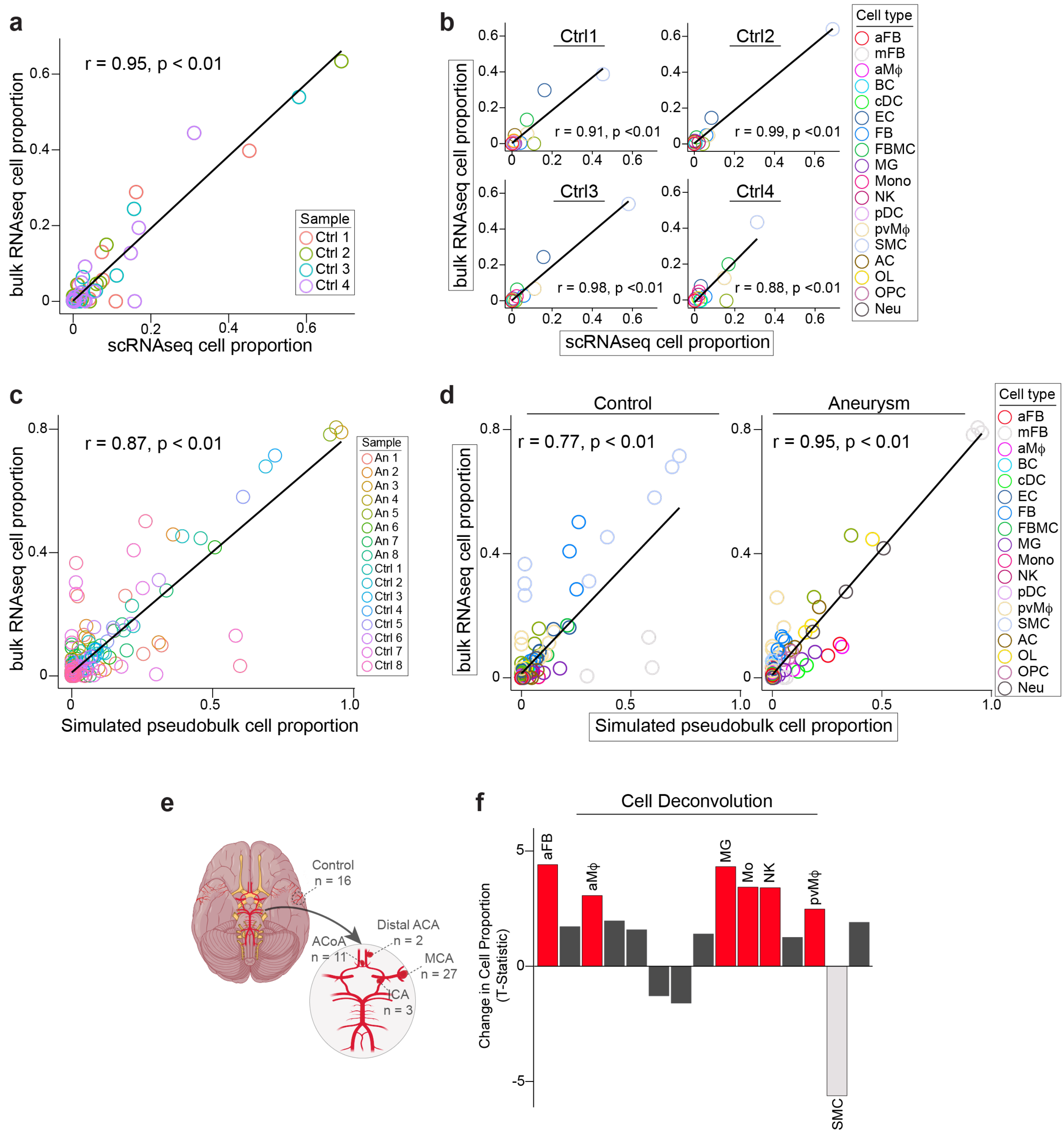

Supplementary Figure 1. Cell-specific composition changes in brain aneurysms. a, Scatter plot of single-cell RNAseq cell proportions (*x*-axis) and deconvoluted bulk RNAseq cell proportions using BayesPrism (*y*-axis). Paired single-cell and bulk RNA sequencing analyses performed on same control cortical vascular samples (n = 4 donors). r, two-sided Pearson correlation coefficient. EC, endothelial cell; SMC, smooth muscle cell; FbM, fibromyocyte; FB, fibroblast; aFB, activated fibroblast; mFB, myofibroblast; TC, T-cell; NK, natural killer cell; BC, B-cell; pDC, plasmacytoid dendritic cell; cDC, conventional dendritic cell; pvMϕ, perivascular macrophage; Mo, monocyte; aMϕ, *APC5*$^{+}$ macrophage; MG, microglia; AC, astrocyte; Neu, neuron; OL, oligodendrocyte; OPC, oligodendrocyte precursor cell. **b,** Scatter plot as described in **a** and faceted by sample. **c-d**, Scatter plots of

scRNAseq cell proportions (*x*-axis) and cell proportions from deconvolution of simulated pseudobulk RNAseq using BayesPrism (*y*-axis) in control cerebrovasculature (n = 8 donors) and brain aneurysms (n = 8 donors). Results shown by sample (**c**) and by condition (**d**). **e**, Schematic showing location distribution and number of donors for samples obtained from prior bulk RNA sequencing. ACoA, anterior communicating artery; ACA, anterior cerebral artery; ICA, internal carotid artery; MCA, middle cerebral artery; PICA, posterior inferior cerebellar artery. Inset shows Circle of Willis. Circles denote location of control samples from cortical vasculature. Dashed lines to aneurysms denote location of aneurysm samples. Created in BioRender. Winkler, E. (2026) https://BioRender.com/kjdamt8. **f**, Bar graph of change in cell population *t*-statistic in bulk RNA sequenced aneurysms following cell deconvolution analysis (n = 16 controls; n = 43 aneurysms). Red denotes statistical enrichment in aneurysms. Gray denotes statistical significant enrichment in controls (FDR < 0.05, two-sided *t*-test, Benjamini-Hochberg correction).

Supplementary Figure 2

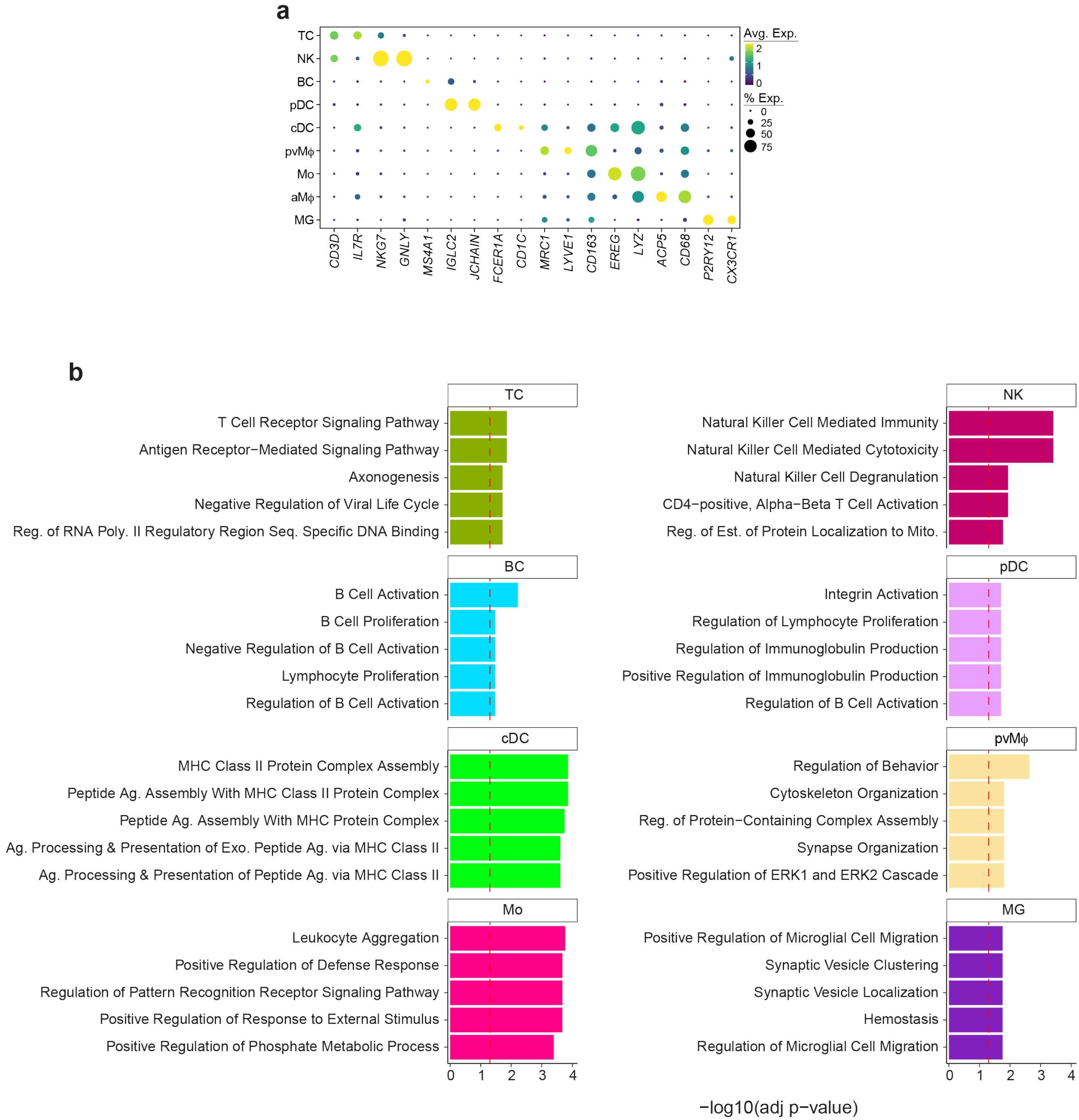

Supplementary Figure 2. **Immune cells in brain aneurysms and controls. a**, Dot plot showing expression of immune cell population marker genes. Avg., average; Exp., expression. Yellow, high expression; blue, low expression. TC, T-cell; NK, natural killer cell; BC, B-cell; pDC, plasmacytoid dendritic cell; cDC, conventional dendritic cell, pvMϕ, perivascular macrophage; Mo, monocyte, aMϕ, *APC5*$^{+}$ macrophage; MG, microglia. **b,** Enriched biological processes from marker genes for each immune subcluster ordered by statistical significance. Benjamini-Hochberg adjusted $P < 0.05$; dashed line, Benjamini-Hochberg adjusted P cutoff of 0.05.

Supplementary Figure 3

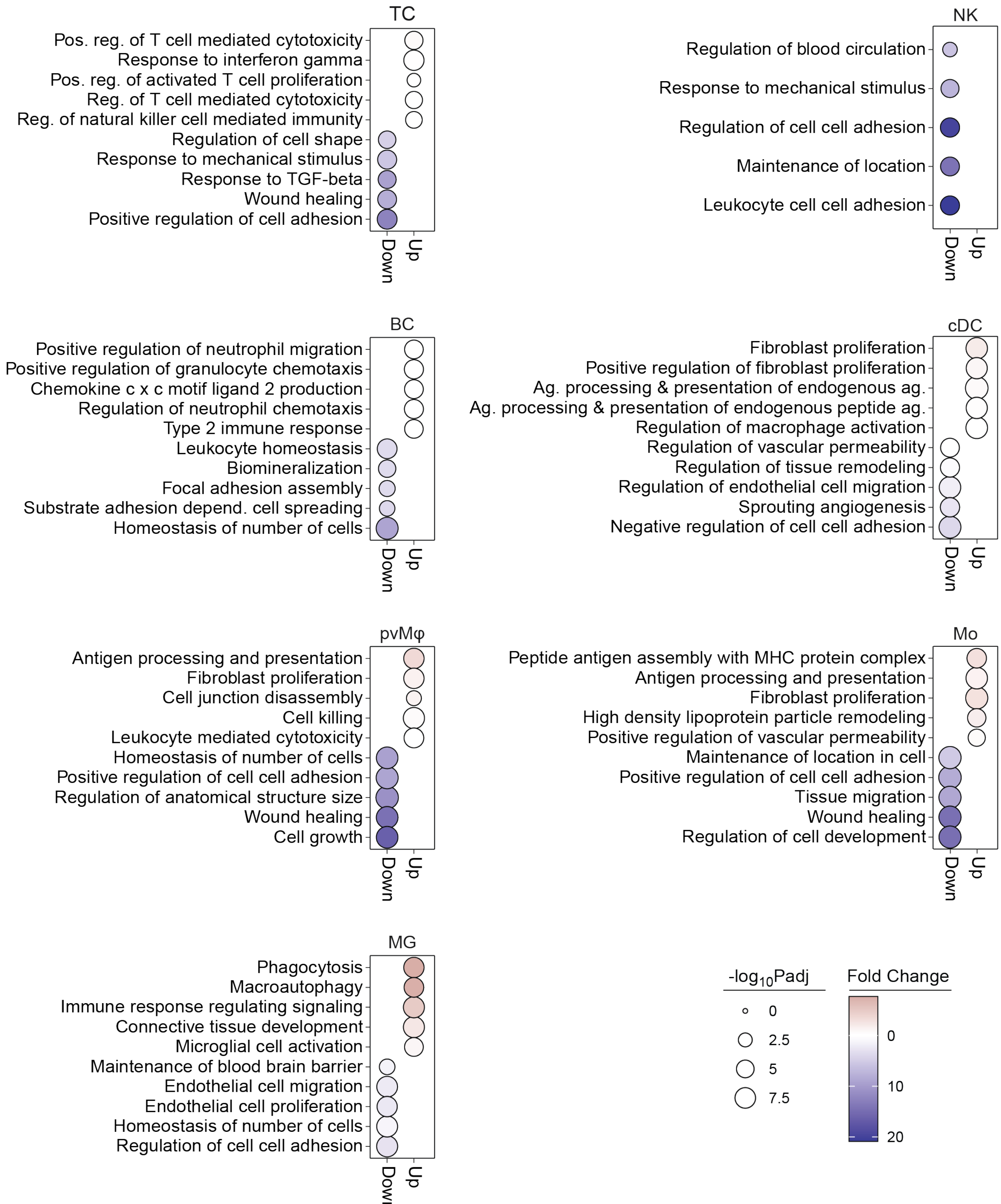

Supplementary Figure 3. Differentially expressed pathways in canonically annotated immune cells. Dotplot of enriched biological process in aneurysmal immune cells. (Bonferroni-corrected p-value < *0.05*, based on Single-Cell Pathway Analysis). Red, upregulated; blue, downregulated. TC, T-cell; NK, natural killer cell; BC,

B-cell; pDC, plasmacytoid dendritic cell; cDC, conventional dendritic cell, pvMϕ, perivascular macrophage; Mo, monocyte; MG, microglia.
