# Supplementary material for: Cerebrovascular vulnerability and fibrosis in human brain aneurysms
Source: Nat Neurosci. 2026 Jun 10;29(8):1814–25. doi: 10.1038/s41593-026-02326-9 (PMC13433319; doi:10.1038/s41593-026-02326-9)
Supplement: Supplementary file 2 [file 41593_2026_2326_MOESM2_ESM.pdf]

nature portfolio

Corresponding author(s): Ethan A. Winkler & Michael T. Lawton

Last updated by author(s): April 16, 2026

Reporting Summary

Nature Portfolio wishes to improve the reproducibility of the work that we publish. This form provides structure for consistency and transparency in reporting. For further information on Nature Portfolio policies, see our Editorial Policies and the Editorial Policy Checklist.

Statistics

For all statistical analyses, confirm that the following items are present in the figure legend, table legend, main text, or Methods section.

n/a	Confirmed	
☐	☒	The exact sample size (*n*) for each experimental group/condition, given as a discrete number and unit of measurement
☐	☒	A statement on whether measurements were taken from distinct samples or whether the same sample was measured repeatedly
☐	☒	The statistical test(s) used AND whether they are one- or two-sided<br>*Only common tests should be described solely by name; describe more complex techniques in the Methods section.*
☐	☒	A description of all covariates tested
☐	☒	A description of any assumptions or corrections, such as tests of normality and adjustment for multiple comparisons
☐	☒	A full description of the statistical parameters including central tendency (e.g. means) or other basic estimates (e.g. regression coefficient) AND variation (e.g. standard deviation) or associated estimates of uncertainty (e.g. confidence intervals)
☐	☒	For null hypothesis testing, the test statistic (e.g. *F*, *t*, *r*) with confidence intervals, effect sizes, degrees of freedom and *P* value noted<br>*Give P values as exact values whenever suitable.*
☐	☒	For Bayesian analysis, information on the choice of priors and Markov chain Monte Carlo settings
☐	☒	For hierarchical and complex designs, identification of the appropriate level for tests and full reporting of outcomes
☐	☒	Estimates of effect sizes (e.g. Cohen's *d*, Pearson's *r*), indicating how they were calculated

Our web collection on statistics for biologists contains articles on many of the points above.

Software and code

Policy information about availability of computer code

Data collection	No software was used for data collection.
Data analysis	STARSolo v2.7.10a; Seurat's SCTransform v2; UCell v2.2.0; Propeller v0.0.3; SCPA v1.6.1; scPagwas v1.3.0; Monocle3 v1.3.4; SeuratWrappers v0.0.3; enrichR v3.2; CellChat v2.1.2; Scissor v2.0.0; BayesPrism v2.2.2; Kallisto v0.46.2; Sleuth v0.30.1; BANKSY v0.1.6; Other bespoke analyses can be found in the attached Github link and software parameters can be found in detail in the Methods section.

For manuscripts utilizing custom algorithms or software that are central to the research but not yet described in published literature, software must be made available to editors and reviewers. We strongly encourage code deposition in a community repository (e.g. GitHub). See the Nature Portfolio guidelines for submitting code & software for further information.

Data

Policy information about availability of data

All manuscripts must include a data availability statement. This statement should provide the following information, where applicable:

- Accession codes, unique identifiers, or web links for publicly available datasets
- A description of any restrictions on data availability
- For clinical datasets or third party data, please ensure that the statement adheres to our policy

Data is available to explore via the interactive web-based cell viewer: https://brain-aneur.cells.ucsc.edu/. Raw sequencing FASTQ files are deposited to dbGAP: phs002624.v6.p1. In addition, custom code utilized for bespoke analyses is available at https://github.com/jwangbio/aneurysm-analysis.

Research involving human participants, their data, or biological material

Policy information about studies with human participants or human data. See also policy information about sex, gender (identity/presentation), and sexual orientation and race, ethnicity and racism.

Reporting on sex and gender	For the single-cell experiments, 9 individuals are male and 7 individuals are female. For the Xenium experiments, 3 individuals are male and 6 individuals are female. For the Slide-Seq spatial atlas, 1 individual is male and 1 individual is female. For the bulk RNA-seq dataset, 21 individuals are male and 38 individuals are female. All of the sex-based metadata is also included in Supplementary Table 1 for the reader to peruse.
Reporting on race, ethnicity, or other socially relevant groupings	Race, ethnicity, or any other socially relevant groupings was not taken.
Population characteristics	The age of the specimens range from 16 y.o to 85 y.o with a median age of 50.5 y.o.
Recruitment	Written informed consent with the purpose of collecting tissues for the purpose of research was obtained from patients prior to the planned neurosurgical operation or post-mortem tissue acquisition. No compensation was given to donors.
Ethics oversight	Institutional review board and ethics committee at each participating site: University of California San Francisco (UCSF), Barrow Neurological Institute/St. Joseph's Medical Center, the University of Washington, and the Helsinki University Hospital.

Note that full information on the approval of the study protocol must also be provided in the manuscript.

Field-specific reporting

Please select the one below that is the best fit for your research. If you are not sure, read the appropriate sections before making your selection.

☒ Life sciences ☐ Behavioural & social sciences ☐ Ecological, evolutionary & environmental sciences

For a reference copy of the document with all sections, see nature.com/documents/nr-reporting-summary-flat.pdf

Life sciences study design

All studies must disclose on these points even when the disclosure is negative.

Sample size	scPower [PMID: 34785648] was utilized to calculate statistical power afforded by the present aneurysm sample sizes. A total of 68 brain aneurysms and 32 control vessels were used in this study for the genomics analyses.
Data exclusions	All samples were included in the analysis. Decisions for exclusions of cells are clearly stated and justified in the methods section.
Replication	Key bioinformatic conclusions were rigorously validated via orthogonal analyses and datasets and experimentally validated using immunofluorescence stainings.
Randomization	Both aneurysms and control vessels were randomized across batch conditions during the processing for single-cell and spatial genomics.
Blinding	Blinding is not relevant to this study as samples are submitted for sequencing. Any variance in data quality becomes corrected through our rigorous array of QC protocol as detailed in our Methods section.

Reporting for specific materials, systems and methods

We require information from authors about some types of materials, experimental systems and methods used in many studies. Here, indicate whether each material, system or method listed is relevant to your study. If you are not sure if a list item applies to your research, read the appropriate section before selecting a response.

Materials & experimental systems

n/a	Involved in the study	
☐	☒	Antibodies
☐	☒	Eukaryotic cell lines
☒	☐	Palaeontology and archaeology
☒	☐	Animals and other organisms
☒	☐	Clinical data
☒	☐	Dual use research of concern
☒	☐	Plants

Methods

n/a	Involved in the study	
☒	☐	ChIP-seq
☒	☐	Flow cytometry
☒	☐	MRI-based neuroimaging

Antibodies

Antibodies used

Goat anti-human podocalyxin (1:100, R&D Systems, Minneapolis, MN); Mouse anti-human calponin-1 (CNN1, 1:100, MilliporeSigma, Burlington, MA); Rabbit anti-human COL1A1 (E8F4L) (1:100, Cell Signaling Technology, Danvers, MA); Mouse anti-human COL1A1 (E3E1X) (1:100, Cell Signaling Technology); Rabbit anti-human periostin (E5F2S) (1:100, Cell Signaling Technology); Mouse anti-human TRAcP (clone 9C5, 1:100, MilliporeSigma); Rabbit anti-human CD68 (D4B9C) (1:100, Cell Signaling Technology); rabbit anti-human Thy1/CD90 (D3V8A) (1:100, Cell Signaling Technology); Donkey anti-goat AlexaFluor 488 (1:100, Thermo Fisher Scientific); Donkey anti-rabbit AlexaFluor 555 (1:100, Thermo Fisher Scientific); Donkey anti-mouse AlexaFluor 647 (1:100, Thermo Fisher Scientific); Biotin-conjugated donkey anti-mouse antibody (1:100, Thermo Fisher Scientific); DyLight 649 conjugated streptavidin (1:100, Vector Laboratories); MIF mouse antibody clone 2A10-4D3 (5 µg/ml, WH0004282M1, Sigma-Aldrich); CD74 rabbit antibody (5 µg/ml, HPA010592, Sigma-Aldrich); CD68 goat antibody (5 µg/ml, PA5-143237, Invitrogen)

Validation

Antibodies were commerically obtained and have been validated by the manufacturer prior to use.

Eukaryotic cell lines

Policy information about cell lines and Sex and Gender in Research

Cell line source(s)

Human Macrophage Primary Cells were obtained commercially from Celprogen. The cells were obtained from human peripheral blood. Sex of the donors is not available (Catalog #36070-01). Human Brain Vascular Smooth Muscle Cells were obtained commercially from ScienCell. These cells were isolated from human brain. Sex of donors is not available (Catalog #1100). Human Brain Vascular Adventitial Fibroblasts were obtained commercially from ScienCell. These cells were isolated from human brain. Sex of donors is not available (Catalog No.1110)

Authentication

All cell lines were authenticated prior to usage.

Mycoplasma contamination

All cell lines were obtained commercially and validated as being Mycoplasma-free. We have used antibiotics in all our culture media and the incubators were cleaned with antimycotic solutions.

Commonly misidentified lines
(See ICLAC register)

Not applicable.

Plants

Seed stocks

Report on the source of all seed stocks or other plant material used. If applicable, state the seed stock centre and catalogue number. If plant specimens were collected from the field, describe the collection location, date and sampling procedures.

Novel plant genotypes

Describe the methods by which all novel plant genotypes were produced. This includes those generated by transgenic approaches, gene editing, chemical/radiation-based mutagenesis and hybridization. For transgenic lines, describe the transformation method, the number of independent lines analyzed and the generation upon which experiments were performed. For gene-edited lines, describe the editor used, the endogenous sequence targeted for editing, the targeting guide RNA sequence (if applicable) and how the editor was applied.

Authentication

Describe any authentication procedures for each seed stock used or novel genotype generated. Describe any experiments used to assess the effect of a mutation and, where applicable, how potential secondary effects (e.g. second site T-DNA insertions, mosiacism, off-target gene editing) were examined.
